# Genomic epidemiological analysis reveals new insights into the resurgence of *Mycoplasma pneumoniae* in China

**DOI:** 10.3389/fcimb.2025.1611519

**Published:** 2025-11-07

**Authors:** Xue Wang, Dandan Zheng, Cheng Gong, Ming Luo, Aihua Li, Xuecong Duan, Chengcheng Wang, Geng Hu, Xuejiao Guan, Fan Yang, Fang Huang, Lihong Chen

**Affiliations:** 1 NHC Key Laboratory of Systems Biology of Pathogens, National Institute of Pathogen Biology, Chinese Academy of Medical Sciences & Peking Union Medical College, Beijing, China; 2 Beijing Center for Disease Prevention and Control, Beijing Academy for Preventive Medicine, Beijing Institute of Tuberculosis Control Research and Prevention, Beijing, China; 3 Key Laboratory of Respiratory Disease Pathogenomics, Chinese Academy of Medical Sciences, Beijing, China; 4 Beijing Research Center for Respiratory Infectious Diseases, Beijing, China

**Keywords:** mycoplasma pneumoniae, macrolide resistance, genomic epidemiological analysis, genetic variation, comparative genomics

## Abstract

**Objectives:**

After coronavirus disease 2019 pandemic restrictions, *Mycoplasma pneumoniae* (*M. pneumoniae*) re-surged widely across the world. This study aimed to determine the genomic epidemiological characteristics of resurging *M. pneumoniae*, which has dominated the respiratory infection outbreak in Beijing, China, since mid-September 2023.

**Methods:**

*M. pneumoniae* samples were collected from patients with acute respiratory-tract infections in Beijing in 2018–2023. A total of 160 *M. pneumoniae* genomes were sequenced via probe-capture-based approach. The genetic features of *M. pneumoniae* were characterized by multilocus sequence typing and comparative genomic analysis.

**Results:**

In total, 160 patients with *M. pneumoniae* infections were enrolled. ST3 (n = 93) and ST14 (n = 65) were the predominant sequence types. The macrolide-resistant mutation rate of ST3 was maintained at 100%, whereas that of ST14 increased rapidly. Comparative genomic analysis revealed 99% to > 99% similarity among the Beijing strains from 2023 when aligned to the reference M129 genome. The major variation occurs in the P1 gene. MAUVE indicated a lack of rearrangement, yet it included four subtype-specific insertions and non-conserved *hsdS* genes. The phylogenetic tree showed that strains from Asia and other world regions clustered into distinct clades, with significant evolutionary differences. Further genomic analyses identified some Asia-dominant genetic variations in genes associated with genome stability, pathogenesis, and drug resistance.

**Conclusions:**

The 2023 outbreak of *M. pneumoniae* was not attributable to a novel variant but stemmed from the resurgence of the pre-existing strains. Our genomic epidemiological findings demonstrated that the endemic strains in different regions exhibit distinct genomic characteristics, associated with genomic stability.

## 1 Introduction

*Mycoplasma pneumoniae* (*M. pneumoniae*) was one of the major pathogens responsible for respiratory infections before the coronavirus disease 2019 (COVID-19) pandemic, with a worldwide incidence of 8.61% in 2017–2020 (Meyer Sauteur et al., 2023). In the COVID-19 pandemic era, nonpharmaceutical interventions against COVID-19 dramatically reduced the transmission of *M. pneumoniae* throughout the world (Meyer Sauteur et al., 2024b). This low-level prevalence of *M. pneumoniae* persisted until an epidemic spread across four continents from the latter half of 2023 (Chen et al., 2024; Gong et al., 2024; Meyer Sauteur et al., 2024a). This round of *M. pneumoniae* epidemics occurred about 4 years after the previous wave in 2019, with a delay of approximately 1 year relative to the typical periodicity of *M. pneumoniae* epidemics (Beeton et al., 2020). During a similar period, Northern China also experienced a surge in childhood pneumonia, beginning at mid-October 2023, which drew the concern of the World Health Organization (WHO). Our previous study based on epidemiological surveillance revealed that this epidemic of pneumonia among children was mainly driven by several common respiratory pathogens, particularly *M. pneumoniae*, rather than by an emerging pathogen (Gong et al., 2024). Recently, Russia reported cases of respiratory disease with hemoptysis and high fever caused by *M. pneumoniae* infections between March and April 2025, causing renewed concern (XINHUANET, 2025).

Macrolide-resistant *M. pneumoniae* (MRMP) poses a severe public health challenge. The Western Pacific region currently exhibits the highest global prevalence of macrolide-resistant MRMP, with rates exceeding 90% in China and 78.5% in South Korea (Hsieh et al., 2022; Lee et al., 2022a; Wang and Luo, 2022). This drug resistance probably led to the epidemic spread of *M. pneumoniae* by prolonging the duration of the illness and eliminating period. By contrast, the proportion of MRMP has been maintained at very low levels in Europe and the Americas (Kim et al., 2022). The rationale behind this difference in the rates of MRMP has yet not been fully clarified, although one widely acknowledged explanation attributes it to the overuse of macrolides in some Asian countries. This perspective seems confirmed in Japan. The proportion of MRMP in Japan peaked at 81.8% in 2012 and has fluctuated and decreased since then, falling to 14.3% in 2018 (Nakamura et al., 2021), which was probably attributed to introducing tosufloxacin in clinical guidelines for treating MRMP, the reduction in antibiotic consumption under the National Action Plan on Antimicrobial Resistance and genotype shift (Ishiguro et al., 2021; Nakamura et al., 2021; Kenri et al., 2023). Even so, the proportion of MRMP is still higher in Japan than in European countries. Notably, the proportion of MRMP in China is increasing, despite the stricter regulation of antibiotic use in recent years (Ministry of Health, 2012; Medical Administration Hospital Authority, 2015) and the National Action Plan for Curbing Bacterial Resistance (Medical Administration Hospital Authority, 2016, 2022). These data indicate that the overuse of macrolides is probably not the only reason for the high proportion of MRMP in Asia. A comparison of the genetic characteristics of *M. pneumoniae* strains in high- and low-MRMP-prevalence regions should extend our understanding of the mechanism underlying this discrepancy.

In this study, 160 *M. pneumoniae* strains identified by the Beijing Respiratory Pathogen Surveillance System in Beijing between 2018 and 2023 were collected and subjected to whole-genome sequencing (WGS). Combining these genomes with a global collection of 430 *M. pneumoniae* genomes available from a public database, we conducted a genomic epidemiological analysis in an attempt to identify whether potential new variants are associated with the resurgence of *M. pneumoniae* in Beijing and to investigate the genomic drivers of the regional differences in the rates of MRMP infections.

## 2 Materials and methods

### 2.1 Samples and publicly available *M. pneumoniae* genomes

Based on the Beijing Respiratory Pathogen Surveillance System, 160 *M. pneumoniae*-positive samples, identified with PCR, were collected from January 2018 to November 2023 (**Supplementary Table 1**). We also downloaded 430 publicly available datasets (accessed on 10/03/2024) from the National Center for Biotechnology Information (NCBI) repository (**Supplementary Table 2**). The M129 (accession: NC_000912.1, P1-type1) and FH (accession: NZ_CP010546.1, P1-type2) were representative of type 1 and type 2 strains, respectively. The M129 was used as the reference genome in our bioinformatic analysis.

### 2.2 *M. pneumoniae* culture

Six representative samples collected in 2023 were cultured individually in 2 ml of *Mycoplasma* color-changing liquid medium (OXOID) at 37°C. Upon observing a color change from red to yellow, 0.1 ml of each suspension was transferred to solid agar plates. *M. pneumoniae* isolates were then purified using dilution techniques. Nucleotide identification for each purified isolate was performed by the real time PCR method, as previously described (Dumke et al., 2007).

### 2.3 Whole-genome sequencing and genome assembly

We obtained the *M. pneumoniae* genome data from 160 clinical specimens. Genomic DNA was extracted from each isolate with the Wizard Genomic DNA Purification Kit (Promega, Madison, WI, USA), according to the manufacturer’s protocol. To enrich the nucleic acids, we used an *M. pneumoniae*-specific hybridization capture probe designed in our laboratory. Library preparation for next-generation sequencing (NGS) was performed using the Enzyme Plus Library Prep Kit (iGeneTech, BJ, BJ, China) and the TargetSeq One Kit (iGeneTech). The libraries were sequenced on the NovaSeq 6000 platform (Illumina, San Diego, CA, USA), generating paired-end reads of 150 bp in length. The sequencing depth for the *M. pneumoniae* strains averaged approximately 1062 X.

6 Beijing representative strains from November 2023 were further selected for long-read sequencing using the Nanopore GridION X5 (Oxford Nanopore). Short-reads were assembled *de novo* using the Geneious prime software (version 2023.2.1; Biomatters Ltd., Auckland, New Zealand). The number of contigs generated ranged from 5 to 14 per strain. Overlapping and joining of the contigs were performed with long-reads. The initial short-reads were aligned to the *de novo* assembled genome for the correction of errors.

### 2.4 SNP and indel calling

We evaluated the quality of the Illumina sequencing reads for the 160 *M. pneumoniae* genomes and trimmed adapter sequences using Trimmomatic v0.3 (Bolger et al., 2014). Clean Valid data were then aligned to the M129 reference genome sequence using the Burrows–Wheeler algorithm, implemented in BWA with the default parameters (Li and Durbin, 2010). After deduplication, we confirmed that the coverage of all genomic sites with a sequencing depth greater than 10 exceeded 95% for all samples. Single-nucleotide polymorphisms (SNPs) and insertions and deletions (indels) were called with a consensus quality score of 30 using the HaplotypeCaller program in GATK v4.1.7 with the local *de novo* assembly of haplotypes in an active region (McKenna et al., 2010). The VariantFiltration program in GATK was used to hard-filter variant calls based on the criteria recommended by the GATK team to generate high-confidence SNPs and indels. The SNPs and indels in the 141 previously published *M. pneumoniae* raw sequence datasets, together with artificial short-read datasets of the 289 published genomes generated by the ART-Illumina (Said Mohammed et al., 2018) read simulation tool, were also called with the aforementioned method. In the analysis of different subtypes in this study, SNPs and indels shared by more than 60% of strains in Asia and by less than 40% of strains in other regions of the world were identified as Asia-dominant SNPs and indels.

### 2.5 Phylogenetic analysis

ICEfinder (Liu et al., 2019) was used to detect integrative and conjugative elements (ICEs) and integrative and mobilizable elements (IMEs), and PHASTER (Arndt et al., 2016) was used to identify prophages. SNPs located within these regions and those in repetitive regions of *M. pneumoniae* M129 were excluded. To avoid the potential homoplasy effects of drug-resistance-associated mutations on phylogeny, SNPs were also excluded from the dataset used for phylogenetic tree construction when they were located in known drug-resistance-related genes, including MPN_r02 (Lucier et al., 1995), MPN_166 (Ou et al., 2015), and MPN_170 (Pereyre et al., 2016). A concatenated superset of SNPs relative to M129 reference genome was generated across all 160 sequenced strains and all 430 published datasets. SNP sites with missing data in any of the strains within the dataset were removed. The refined SNP set was used to construct a maximum-likelihood phylogeny with RAxML (Stamatakis, 2014), using the GTRgamma substitution model. Node reliability was assessed with a bootstrap analysis of 100 resampled datasets. The iTOL server (Letunic and Bork, 2024) was used for the manipulation and presentation of the phylogenetic trees.

### 2.6 Molecular typing

Multilocus sequence typing (MLST) was performed with the MLST scheme specific for *M. pneumoniae* available at the PubMLST database (Jolley et al., 2018). Eight housekeeping genes were analyzed by aligning their sequences against the references on PubMLST, and sequence types (STs) were assigned based on the allele profiles of these genes.

### 2.7 Comparative genomics

Completed genomes were aligned to the reference M129 genome using BRIG (Alikhan et al., 2011) to visualize overall sequence similarity between the strains. MAUVE was used to detect large chromosomal rearrangements, deletions, and duplications (Darling et al., 2004). M129 and FH were used as typical strains for type 1 and type 2.

### 2.8 Statistical analysis

Median values and interquartile ranges were calculated for continuous variables, and percentages were used for categorical variables. Comparisons of different groups were analyzed with the χ2 test or Fisher’s exact test. Significance testing was performed by redistributing the mutation events in each gene in different groups and deriving an empirical p value. The q values for the discovery rates were then calculated to account for multiple hypothesis testing, using the Benjamini–Hochberg procedure (Benjamini and Hochberg, 1995).

## 3 Results

### 3.1 Strain characteristics

**Figure 1:**
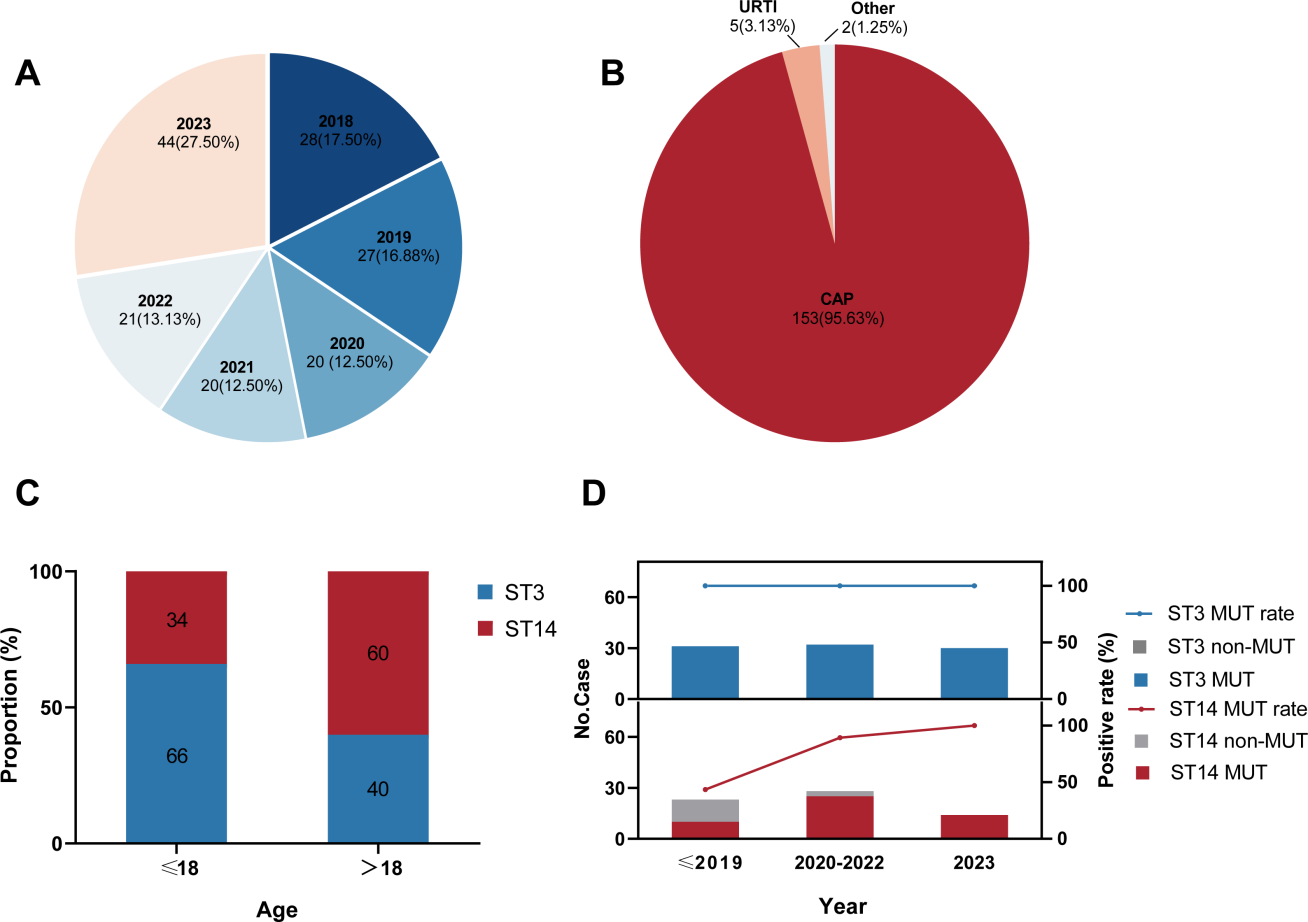
Characteristics of 160 *Mycoplasma pneumoniae* strains included in this study. **(A)** Distribution of the collection years. **(B)** Distribution of the patients’ clinical symptoms. CAP: community-acquired pneumonia; URTI: upper respiratory-tract infection. **(C)** ST distribution in different age groups. ST: sequence type. **(D)** Macrolide-resistance mutations in 23S rRNA among strains by ST. MUT: macrolide-resistance mutation in the 23S rRNA gene.

A total of 160 *M. pneumoniae* genomes of patients with *M. pneumoniae* infection in Beijing in 2018–2023 were included in the current genomic study (**Figure 1A**; **Supplementary Table 1**). Strains from patients with community-acquired pneumonia accounted for 95.6% (**Figure 1B**), including 28 patients with severe CAP. Male patients accounted for 50.6%, demonstrating a balanced sex ratio in the cohort. The median age was 8 years (P25, 6 years; P75, 27.5 years). An analysis identified four sequence types (STs): predominantly ST3 (n = 93; 58.2%) and ST14 (n = 65; 40.6%), with ST2 (n = 1; 0.6%) and ST17 (n = 1; 0.6%) as minor types. Interestingly, the ST distributions were significantly different (χ2 = 9.243, P < 0.01) among the age groups in this study, with ST3 predominant in children and ST14 prevalent in adults (**Figure 1C**).

Based on our genomic analysis, the total proportion of MRMP in Beijing from 2018–2023 was 88.8% (142 of 160; **Supplementary Table 1**), all of which possessed the A2063G resistance mutation in domain V of 23S rRNA gene. However, the rate of macrolide-resistance mutation varied in the major ST groups. The macrolide-resistant mutation rate in the ST3 strains was maintained at 100%, whereas the overall mutation rate among the ST14 strains was 75.4% (**Supplementary Table 1**), showing a rapidly rising trend in recent years (**Figure 1D**). Specifically, within the ST14 strains, MRMP accounted for 43.5% before 2020, increased significantly to 89.3% in 2020–2022, and then reached a surprising 100% in 2023. But no ST2 or ST17 strain exhibited resistance to macrolides in this study.

### 3.2 Phylogenetic associations

**Figure 2:**
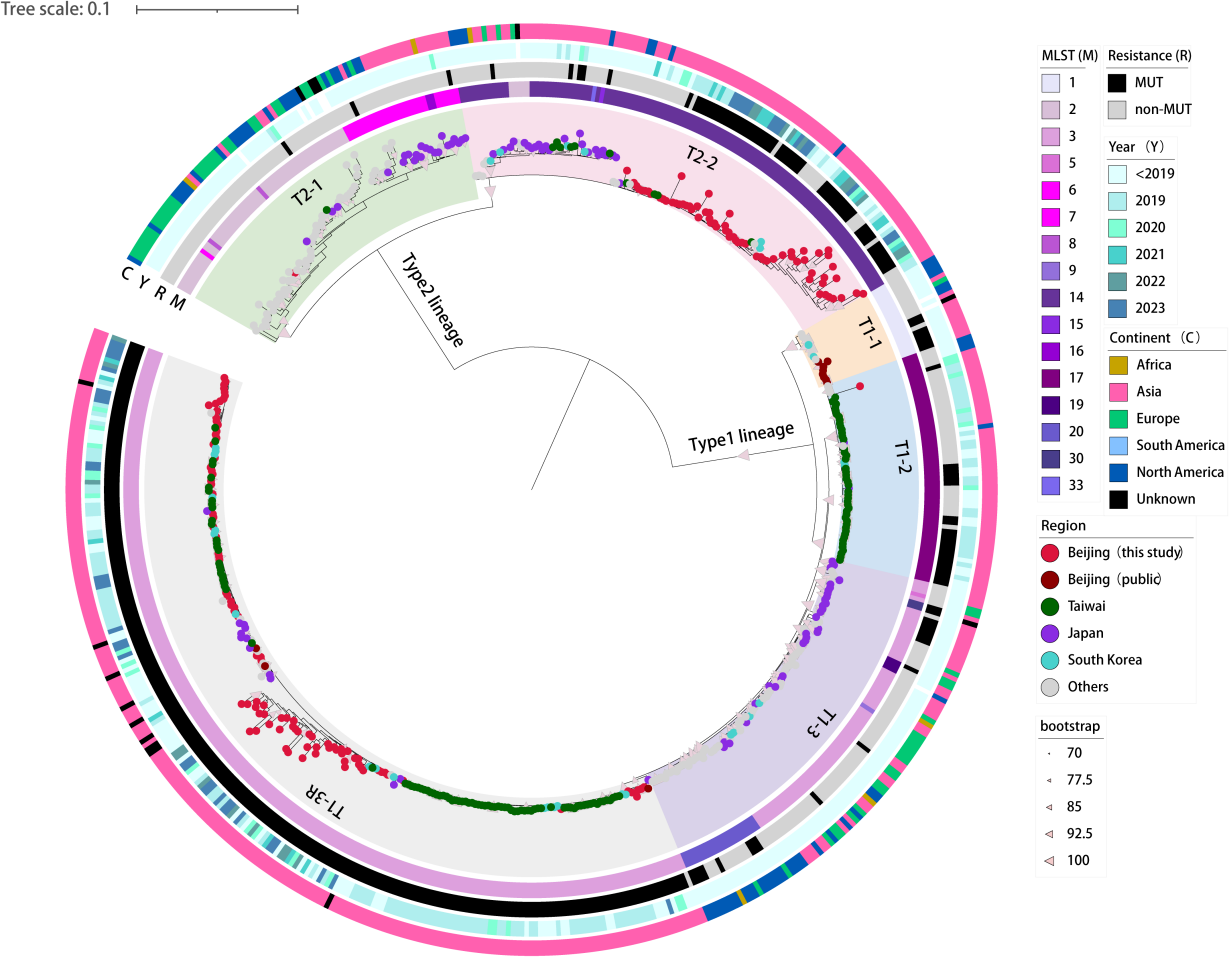
Phylogenetic tree based on genome-wide SNPs in the 160 newly sequenced strains and 430 *M. pneumoniae* genomes available from public domain. A maximum-likelihood phylogenic tree constructed with RAxML using the GTRgamma substitution model. The tree was rooted on the midpoint. Strips are color-coded from inner to outer by ST group, 23S rRNA mutation, isolation year, and continent (key), respectively. Tree scale represents substitutions per site.

Based on the 5707 SNPs present in the core genomes, we constructed a genome-wide phylogenetic tree of the 590 *M. pneumoniae* strains, including 160 strains from this study and 430 global genomes available from public database (**Figure 2**; **Supplementary Figure S1**). As expected, the phylogeny of *M. pneumoniae* formed two major clades in accordance with the P1 typing and could be further divided into six subclades, consistent with previous results (Hsieh et al., 2022; Kenri et al., 2023; Kubota et al., 2025). The p1-type 1 clade was divided into four subclades T1-1, T1-2, T1–3 and T1-3R, and the p1-type 2 clade into subclades T2–1 and T2-2. In general, the STs of the 590 strains were consistent with the phylogenetic groups. Subclades T1-1, T1–2 and T1-3R consisted solely of ST1, ST17 and ST3 strains, respectively. However, the large subclades always included multiple STs. Major ST of Subclade T1–3 was ST3 with ST5, ST9, ST19, ST20, and ST30. Subclade T2–1 consisted of ST2, ST6, ST7, ST8, and ST16. Subclade T2–2 showed a high proportion of ST14 strains followed by ST2, ST15, and ST33.

A phylogenetic analysis showed that 160 *M. pneumoniae* strains from Beijing largely grouped into subclades T1-3R and T2-2, two major Asia-prevalent subclades within the tree (**Figure 2**). Among the two subclades, the 160 Beijing strains and those isolated from Asia have relatively close genetic locations. Moreover, no correlation between the phylogenetic groups and the year of isolation was observed in Beijing strains.

A spatial analysis revealed that the subclades T1-2, T1-3R and T2–2 were predominantly consisted of strains from Asian countries. On the contrary, strains from other regions of the world (e.g., Europe, America, and Africa) generally formed distinct groups distant from the Asian strains. Specifically, subclades T2–1 and T1–3 were dominated by *M. pneumoniae* strains collected from regions outside Asia, with only a few exceptions of Japan strains (**Figure 2**).

### 3.3 Genome comparison

**Figure 3:**
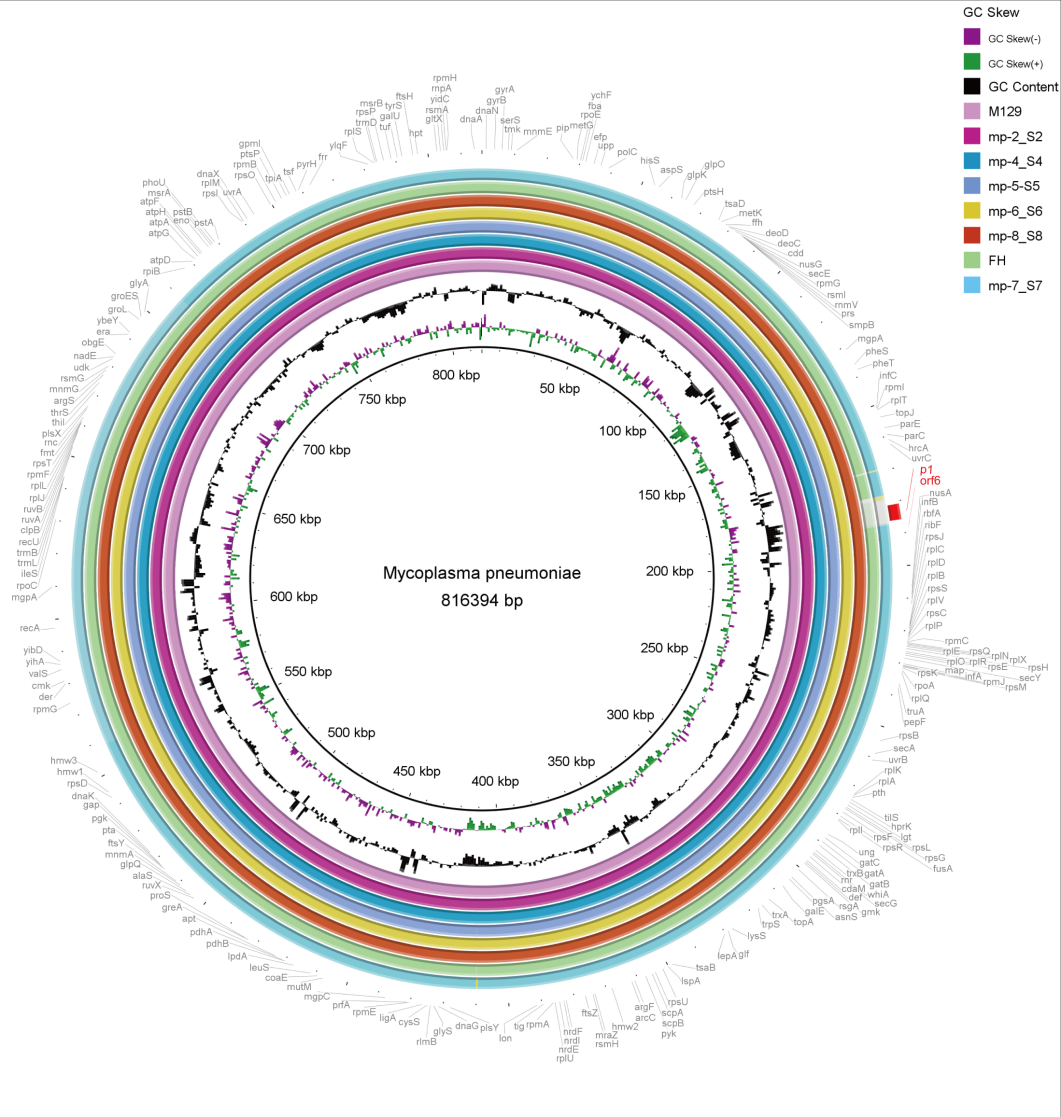
Overall sequence identity of the 6 sequenced strains, M129 and FH strain with the reference M129 genome. Solid coloration indicates > 99% identity and transparent grey indicates approximately 95% identity. Location in the reference genome is indicated by numeration on the inside of the ring. GC skew is a measure of the relative content of G and C. Purple bars signify positive GC skew (+), whereas green bars denote negative GC skew (-). GC content in the reference genome is indicated by the black bar graphs (bars pointing toward the outside of the circle indicate high GC content). Annotated genes of the reference genome are shown outside the ring. As typical strains, M129 associates with type 1 and FH links to type 2.

The 6 completed genomes of Beijing representative strains were aligned using a variety of methods. To determine their overall similarity, the genomes were aligned to the reference M129 genome using BRIG, a BLAST-based alignment method. Overall, the genomes were 99% to > 99% identical. The similarity dropped to approximately 95% in the type 2 strains, where the *p1* gene (MPN141) and the *orf6* gene (MPN142) harbored (**Figure 3**).

**Figure 4:**
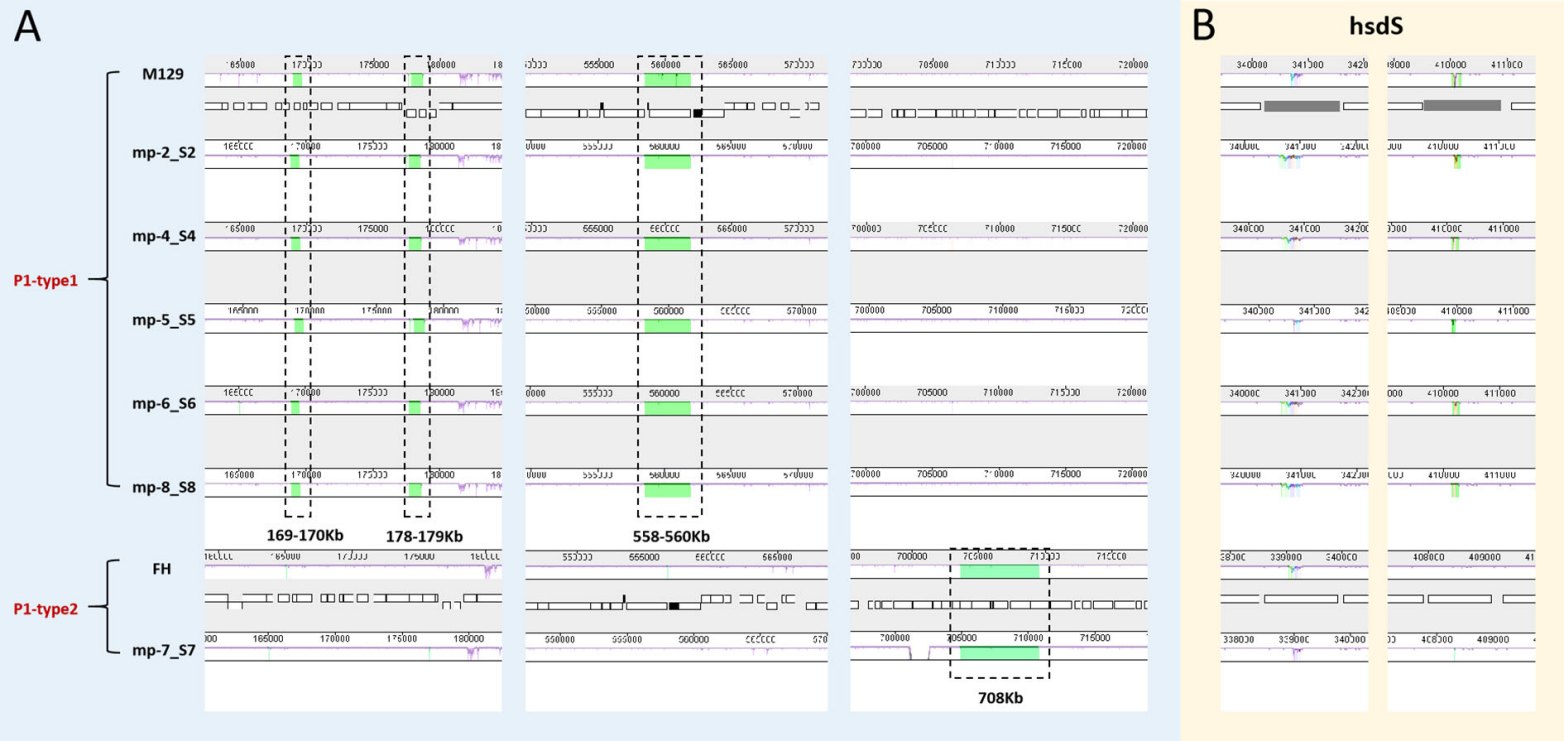
Whole genome alignment of the 6 sequenced strains with 2 reference sequences using MAUVE. Regions no-colored in MAUVE are conserved across all strains. Differently colored blocks are conserved in some strains. Open boxes indicate the location of genes. All positions and annotations are based on M129 strain. M129 and FH are typical strains of type 1 and 2, respectively. **(A)** Close up of 4 subtype-specific insertions. **(B)** Close up of non-conserved structural regions within *hsdS* genes (mpn285 and mpn289). *hsdS*: type I restriction-enzyme-like protein S subunit.

We also aligned the genomes using MAUVE to detect large chromosomal rearrangements, deletions, and duplications. All genomes fell into three conserved locally collinear blocks (LCBs), which were in the same order without any rearrangement. MAUVE detected four subtype-specific insertions according to M129 numbering: three type 1-specific insertions (169–170 kb, 178–179 kb, and 558–560 kb) and a type 2-specific insertion (located at 708 kb). *HsdS* genes (encoding a type I restriction-enzyme-like protein S subunit) were considered to be associated with non-conserved structural regions (**Figure 4**).

### 3.4 Asia-dominant genetic variations

**Figure 5:**
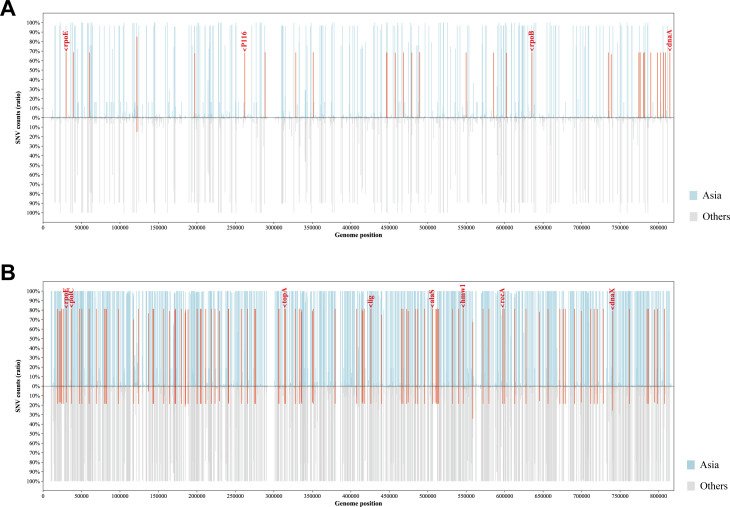
Profiles of Asia-dominant SNPs. The proportion and genomic distribution of all SNP in p1-type 1 strains **(A)** and p1-type 2 strains **(B)**. In the top plot, the proportions of SNPs in Asian strains at each position are plotted as a light blue bar graph; and in the bottom plot, those in strains from other regions of the world are plotted as a light gray bar graph. Positions of Asia-region-dominant SNPs are indicated in red.

To establish the genetic basis of these distinct phylogenetic features of the Asian *M. pneumoniae* strains, we screened for potential Asia-dominant SNPs and indels based on our dataset (**Figure 5**). The Asia-dominant variations of the p1-type 1 strains were distributed in 29 genes and four intergenic regions. Nonsynonymous sequence variations were found in 22 protein-coding genes. Among these, *dnaA* (encoding the chromosomal replication initiator protein DnaA, MPN686) is associated with genome stability. Moreover, three protein-coding genes were annotated as potential virulence factors: *rpoE* (encoding a DNA-directed RNA polymerase delta subunit, MPN024), P116 gene (encoding the adhesion protein P116, MPN213), and *rpoB* (encoding an RNA polymerase beta subunit, MPN516). The Asia-dominant variations in the p1-type 2 strains were distributed in 78 genes and 21 intergenic regions. Nonsynonymous sequence variations were found in 46 protein-coding genes. Six genes were associated with genome stability: *polC* (encoding a DNA polymerase III alpha chain, MPN034), *topA* (encoding a DNA topoisomerase, MPN261), *lig* (encoding a DNA ligase, MPN357), *recA* (encoding a recombination protein RecA, MPN490), *dnaX* (encoding a complex ATPase, MPN618), and *hsdR* (encoding a type I restriction-enzyme-like protein R subunit, MPN345). Three genes were potentially involved in virulence: *rpoE*, *alaS* (encoding an alanyl-tRNA synthetase, MPN419), and *hmw1* (encoding a cytadherence accessory protein HMW1, MPN447). A full list of the Asia-dominant SNPs and indels identified are shown in **Supplementary Table 3**.

## 4 Discussion

This study revealed different distributions of STs across age groups in patients with *M. pneumoniae* infections and disparities in the drug-resistance rates among the STs prevalent in Beijing. Of the two main STs of the Beijing *M. pneumoniae* strains, adult patients were more commonly infected with ST14 strains, whereas ST3 strains were predominant in children. This is consistent with a previous study in Taiwan (Hung et al., 2021). ST3 (93/94) and ST14 (65/66) were the predominant types of p1-type 1 and 2, respectively. The correlation between the *p1* types of *M. pneumoniae* infections and age group implies that the older population may have pre-existing immunity to the p1-type 1 strains. In this study, the Beijing strains showed a high prevalence of MRMP (88.8%) in 2018–2023, with varying resistance rates among different STs. Strikingly, 100% of the ST3 strains showed MRMP, whereas the proportion of MRMP in the ST14 strains increased dramatically from 43.5% to 100% during the last 6 years. In contrast to other regions of Asia, the shift in prevalent STs occurred between 2002–2016 (ST3 and ST14) and 2018–2019 (ST7 and ST33) in Japan, resulting in a rapid drop in the resistance rate to 11.3% in 2018–2019 (Morozumi et al., 2020). However, some researchers have questioned the validity of ST33. The ST33 and ST14 strains exhibit only a single SNP difference in the *adk* marker of the MLST scheme, which notably occurs within the primer sequence of the original MLST method. Caution is needed when interpreting MLST differences in the phylogenetic classification of *M. pneumoniae* strains. Otherwise, slight genetic variations may overestimate the phylogenetic relationship between strains (Kubota et al., 2025). Therefore, we hypothesize that there is a correlation between the dominant ST groups and the macrolide-resistance rate. The driving force behind the shift in prevalent STs requires further investigation.

Analysis of spatial genomic epidemiology reveals clear differences between *M. pneumoniae* genomes in Asia and those from other world regions. In this study, endemic strains from Asia and other world regions clustered in distinct clades on a phylogenetic tree. The differences in geographic distributions may indicate ongoing divergence within the species or imply possible variations in the patterns of transmission. The varying proportions of MRMP in different geographical regions also provides strong evidence of spatial genomic differences. The highest proportion of MRMP infections was 53.4% in the western Pacific region, followed by 9.8% in Southeast Asia, 8.4% in the Americas, and 5.1% in Europe from 2000 to 2019 (Kim et al., 2022).

It is widely accepted that variations in antibiotic resistance rates across regions are associated with local strategies for drug treatment (Morozumi et al., 2020; Nakamura et al., 2021). Nevertheless, it is also possible that the genomes of the Asian endemic *M. pneumoniae* strains have an inherently higher propensity for mutations than those in other regions. This could be an overlooked genetic factor contributing to drug resistance. Our data show that the Asia-dominant mutations were associated with multiple genes involved in bacterial replication. It is reasonable to infer that mutations in *dnaA* (Washington et al., 2017) may affect genome integrity and regulation of DNA damage. The M1376I mutation in PolC (Sanders et al., 2010) may lead to changes in the fidelity of replication, thereby increasing the probability of genetic mutations. Mutations in genes encoding replication enzymes, such as *topA*, *lig* and *dnaX* imply potential changes in both the bacterial growth rate and genetic mutation rates in the group of Asian-endemic strains. RecA repairs broken chromosomes by homologous recombination to maintain the integrity of the genome (Singh et al., 2010). The insertion in *hsdR*, which protects bacteria from invasion by exogenous DNA (Obarska-Kosinska et al., 2008), causes a frameshift mutation, although the phenotypic impact of this genetic mutation remains unclear. Taken together, genetic variations in *M. pneumoniae* genes associated with replication could lead to changes in replicase fidelity, gene repair, and gene recombination, which may contribute to the increased genomic mutation rate in the Asian-endemic strains. The Asia-dominant mutations were also closely related to changes in the drug resistance and virulence of *M. pneumoniae*. The changes of *rpoE* (Flannagan and Valvano, 2008), *rpoB* (Ishikawa et al., 2006) and *alaS* (Jovanovic et al., 1999) may affect the drug-induced stress response mechanism, rifampin resistance and novobiocin resistance in *M. pneumoniae*, respectively. Furthermore, mutations in known virulence factors, such as P116 (Sprankel et al., 2023) and HMW1 (Krause et al., 2018), may affect the virulence and adhesion of *M. pneumoniae*. Whether the Asia-dominant mutations in this study impose potential fitness on *M. pneumoniae* circulating in Asia, would be an interesting focus for future studies.

The upsurge in respiratory illnesses among children in northern China in 2023 has attracted global attention. Our previous results demonstrated that the increase in the incidence of respiratory infections in Beijing was mainly attributable to the circulation of multiple known pathogens, primarily *M. pneumoniae* (Gong et al., 2024). However, the role of any potential new variants in the current *M. pneumoniae* outbreak remains unclear. Our temporal genomic epidemiological analysis of the Beijing strains collected from 2018 to 2023 revealed their dispersal across the phylogenetic tree without forming year-related clustering branches. This indicated the extraordinary stability of the Beijing *M. pneumoniae* genome over time, which showed limited divergence. Our findings are largely consistent with a recent report from southern China (Suzhou), which identified two primary epidemic clones, EC1 (in P1-1) and EC2 (in P1-2) (Li et al., 2024). In our cohort, all ST3 genomes and the majority of ST14 genomes clustered within EC1 and EC2, respectively. The observation of this consistent molecular epidemic pattern across southern and northern China suggests a nationwide dissemination of the EC1 and EC2 clonal groups. However, our study provides further nuance regarding the composition of EC2. Whereas Li et al (Li et al., 2024). considered EC2 to be universally macrolide-resistant due to the A2063G mutation in 23S rRNA, our temporal analysis revealed a more complex dynamic. We found that numerous ST14 strains isolated between 2018 and 2020 lacked macrolide-resistance mutations and did not cluster with EC2. In contrast, those from 2021 to 2023 did carry the mutation and belong to EC2. This indicates that EC2 is a specific, macrolide-resistant subset of the broader ST14 population, and its expansion is likely the primary driver of the increased frequency of MRMP in p1-type 2 strains reported in China since 2020 (Jiang et al., 2023; Chen et al., 2024; Gong et al., 2024).

All 6 Beijing representative strains isolated from the 2023 epidemic season were selected for comparative genome analysis. An overall high degree of sequence similarity was found among the strains (99% to > 99% identical to M129 reference genome). BRIG clearly distinguished P1 types 1 and 2. The differences between two subtypes were concentrated to specific areas of the genome, rather than being evenly distributed. This indicates that specific genes encoding proteins involved in host cell interaction, such as the P1 adhesin, are subject to positive selection pressure. MAUVE demonstrated that no large-scale rearrangements were observed among *M. pneumoniae* isolates. The four subtype-specific insertions were consistent with previous reports by Lee et al. Consistent with previous reports (Xiao et al., 2015; Diaz et al., 2017; Lee et al., 2019), we identified subtype-specific insertions that were unique to either type 1 or type 2 isolates. However, discrepancies were noted among these studies regarding the size of subtype-specific insertions and the detection of small insertions. These differences are likely due to genomic variance among *M. pneumoniae* strains isolated across distinct geographic locations and time periods. The non-conserved regions within *hsdS* have been described in this study. The M129 genome had 10 copies of *hsdS* scattered throughout the genome, encoding the DNA sequence specificity (S) subunit of the type I restriction and modification (R-M) enzymes which protect bacteria from invading foreign DNA. Multiple *hsdS* genes with variable tandem repeat (TR) numbers were found in a previous study (Xiao et al., 2015). The gain or loss of TR units change the target specificity. Hence, the presence of variable *hsdS* genes within the genome implies potential epigenetic mechanisms governing gene regulation. (Lee et al., 2022b). described the association of TR number variabilities in *hsdS* with macrolide resistance in *M. pneumoniae*. These results indicate that the current outbreak in Beijing is probably attributable to the local circulation of the original strains, rather than to any new variant(s) of *M. pneumoniae*.

This study has several limitations. First, potential biases may have been introduced by the composition of the genomic dataset used for phylogenetic analysis. The dataset was imbalanced, with fewer non-Asian (n=151) than Asian strains (n=439). Furthermore, a significant temporal discrepancy existed, as the non-Asian strains were collected over an earlier period (1954–2014) compared to the more recent Asian strains. This disparity in sample size and collection timeline may confound the identification of Asian-specific genetic features in real world. Second, our analysis was restricted to genotypic screening for mutations associated with macrolide resistance, without accompanying phenotypic susceptibility testing to confirm the resistance status. However, these limitations did not change the major findings of this study.

In summary, based on a comprehensive genomic epidemiological analysis, our results indicate that the genomes of *M. pneumoniae* strains circulating in Beijing show a high degree of stability. Theoretically, the current high prevalence of *M. pneumoniae*, which began in autumn of 2023, is probably not due to new emerging variants, and is likely to persist over forthcoming years. Compared with regions of low MRMP prevalence, strains from Beijing and other high-MRMP-prevalence regions carried Asia-dominant mutations in genes associated with genome replication and repair, which probably led to higher mutation probability against selection pressure exerted by antibiotics use. Our data provides a baseline for assessing the impact of future interventions and highlight the need for monitoring *M. pneumoniae* variants to reduce the pressure on healthcare resources.

## Data Availability

The datasets presented in this study can be found in online repositories. The names of the repository/repositories and accession number(s) can be found in the article/**Supplementary Material**. The WGS data has been submitted to GSA database of the National Genomics Data Center-Genome Sequence Archive database with accession number CRA017995. The complete genomes were deposited in NCBI database under the accession numbers CP180367-CP180371, CP180517.
