# Supplementary material for: Genomic epidemiological analysis reveals new insights into the resurgence of Mycoplasma pneumoniae in China
Source: Front Cell Infect Microbiol. 2025 Nov 7;15:1611519. doi: 10.3389/fcimb.2025.1611519 (PMC12634620; doi:10.3389/fcimb.2025.1611519)
Supplement: Supplementary file 1 [file DataSheet1.pdf]

Supplementary Material

Data Availability Statement

The WGS data has been submitted to GSA database of the National Genomics Data Center-Genome Sequence Archive database with accession number CRA017995. The complete genomes were deposited in NCBI database under the accession numbers CP180367-CP180371, CP180517.

Supplementary Figures and Tables

1.1 Supplementary Tables

Supplementary Table 1. Metadata of Beijing *Mycoplasma pneumoniae* strains.

CAP: community-acquired CAP, SCAP: severe community-acquired CAP, URTI: upper respiratory tract infection, non-MUT: non-mutation.

ID	Year	Month	Region	Continent	Clinical symptoms	Gender	Age	23S rRNA mutations	ST	P1 subtype	Phylogenetic tree subclade
M1_FKDL210362652-1a	2018	1	China_Beijing	Asia	CAP	female	15	A2063G	3	P1-1	T1-3R
M2_FKDL210362653-1a	2018	1	China_Beijing	Asia	SCAP	male	3	A2063G	3	P1-1	T1-3R
M3_FKDL210362654-1a	2018	1	China_Beijing	Asia	CAP	male	8	A2063G	3	P1-1	T1-3R
M4_FKDL210362655-1a	2018	1	China_Beijing	Asia	URTI	female	11	A2063G	14	P1-2	T2-2
M5_FKDL210362656-1a	2018	1	China_Beijing	Asia	SCAP	female	5	A2063G	14	P1-2	T2-2
M6_FKDL210362657-1a	2018	1	China_Beijing	Asia	CAP	female	8	A2063G	3	P1-1	T1-3R
M7_FKDL210362658-1a	2018	1	China_Beijing	Asia	CAP	male	10	A2063G	3	P1-1	T1-3R
M8_FKDL210362659-1a	2018	1	China_Beijing	Asia	CAP	female	9	non-MUT	14	P1-2	T2-2
M9_FKDL210362660-1a	2018	1	China_Beijing	Asia	CAP	male	7	A2063G	3	P1-1	T1-3R
M10_FKDL210362661-1a	2018	2	China_Beijing	Asia	CAP	female	6	A2063G	3	P1-1	T1-3R
M11_FKDL210362662-1a	2018	2	China_Beijing	Asia	CAP	female	8	A2063G	3	P1-1	T1-3R
M13_FKDL210362664-1a	2018	2	China_Beijing	Asia	CAP	male	27	A2063G	3	P1-1	T1-3R
M15_FKDL210362666-1a	2018	2	China_Beijing	Asia	CAP	female	38	non-MUT	2	P1-2	T2-1
M16_FKDL210362667-1a	2018	2	China_Beijing	Asia	CAP	male	25	non-MUT	14	P1-2	T2-2
M18_FKDL210362669-1a	2018	2	China_Beijing	Asia	CAP	female	50	A2063G	3	P1-1	T1-3R
M19_FKDL210362670-1a	2018	2	China_Beijing	Asia	SCAP	female	11	A2063G	3	P1-1	T1-3R

M20_FKDL210362671-1a	2018	2	China_Beijing	Asia	CAP	male	12	A2063G	3	P1-1	T1-3R
M21_FKDL210362672-1a	2018	2	China_Beijing	Asia	CAP	male	32	non-MUT	14	P1-2	T2-2
M22_FKDL210362673-1a	2018	2	China_Beijing	Asia	CAP	male	11	A2063G	3	P1-1	T1-3R
M23_FKDL210362674-1a	2018	3	China_Beijing	Asia	CAP	male	8	A2063G	3	P1-1	T1-3R
M24_FKDL210362675-1a	2018	3	China_Beijing	Asia	CAP	female	36	non-MUT	14	P1-2	T2-2
M25_FKDL210362676-1a	2018	3	China_Beijing	Asia	CAP	female	30	non-MUT	14	P1-2	T2-2
M26_FKDL210362677-1a	2018	3	China_Beijing	Asia	CAP	male	24	non-MUT	14	P1-2	T2-2
M28_FKDL210362679-1a	2018	3	China_Beijing	Asia	CAP	female	29	non-MUT	14	P1-2	T2-2
M30_FKDL210362681-1a	2018	3	China_Beijing	Asia	CAP	female	31	non-MUT	14	P1-2	T2-2
M31_FKDL210362682-1a	2018	3	China_Beijing	Asia	CAP	male	44	A2063G	3	P1-1	T1-3R
M32_FKDL210362683-1a	2018	3	China_Beijing	Asia	CAP	male	16	A2063G	3	P1-1	T1-3R
M33_FKDL210362684-1a	2018	3	China_Beijing	Asia	CAP	male	33	A2063G	3	P1-1	T1-3R
M34_FKDL210362685-1a	2019	2	China_Beijing	Asia	CAP	female	4	A2063G	3	P1-1	T1-3R
M35_FKDL210362686-1a	2019	2	China_Beijing	Asia	CAP	female	34	A2063G	3	P1-1	T1-3R
M36_FKDL210362687-1a	2019	2	China_Beijing	Asia	CAP	female	31	A2063G	3	P1-1	T1-3R
M37_FKDL210362688-1a	2019	2	China_Beijing	Asia	CAP	female	34	non-MUT	14	P1-2	T2-2
M38_FKDL210362689-1a	2019	2	China_Beijing	Asia	SCAP	female	9	A2063G	3	P1-1	T1-3R
M39_FKDL210362690-1a	2019	2	China_Beijing	Asia	SCAP	female	7	A2063G	14	P1-2	T2-2
M40_FKDL210362691-1a	2019	2	China_Beijing	Asia	SCAP	male	11	A2063G	3	P1-1	T1-3R
M42_FKDL210362693-1a	2019	2	China_Beijing	Asia	CAP	male	6	A2063G	3	P1-1	T1-3R
M43_FKDL210362694-1a	2019	2	China_Beijing	Asia	CAP	female	6	A2063G	3	P1-1	T1-3R
M44_FKDL210362695-1a	2019	2	China_Beijing	Asia	CAP	male	45	non-MUT	14	P1-2	T2-2
M45_FKDL210362696-1a	2019	2	China_Beijing	Asia	CAP	female	46	A2063G	3	P1-1	T1-3R
M46_FKDL210362697-1a	2019	2	China_Beijing	Asia	CAP	female	22	non-MUT	14	P1-2	T2-2
M47_FKDL210362698-1a	2019	2	China_Beijing	Asia	CAP	male	63	A2063G	14	P1-2	T2-2
M48_FKDL210362699-1a	2019	3	China_Beijing	Asia	CAP	female	34	A2063G	3	P1-1	T1-3R
M53_FKDL210362704-1a	2019	2	China_Beijing	Asia	SCAP	female	15	A2063G	3	P1-1	T1-3R
M55_FKDL210362706-1a	2019	3	China_Beijing	Asia	CAP	male	0.25	A2063G	14	P1-2	T2-2
M56_FKDL210362707-1a	2019	3	China_Beijing	Asia	CAP	female	8	A2063G	3	P1-1	T1-3R
M57_FKDL210362708-1a	2019	3	China_Beijing	Asia	CAP	male	46	A2063G	14	P1-2	T2-2
M58_FKDL210362709-1a	2019	3	China_Beijing	Asia	CAP	female	39	non-MUT	14	P1-2	T2-2
M59_FKDL210362710-1a	2019	4	China_Beijing	Asia	CAP	male	69	A2063G	3	P1-1	T1-3R
M60_FKDL210362711-1a	2019	3	China_Beijing	Asia	SCAP	female	6	A2063G	14	P1-2	T2-2
M62_FKDL210362713-1a	2019	5	China_Beijing	Asia	CAP	male	32	non-MUT	14	P1-2	T2-2
M63_FKDL210362714-1a	2019	5	China_Beijing	Asia	CAP	male	8	A2063G	14	P1-2	T2-2
M64_FKDL210362715-1a	2019	5	China_Beijing	Asia	SCAP	male	3	A2063G	14	P1-2	T2-2
M65_FKDL210362716-1a	2019	5	China_Beijing	Asia	SCAP	male	7	A2063G	3	P1-1	T1-3R
M66_FKDL210362717-1a	2019	8	China_Beijing	Asia	SCAP	female	8	A2063G	14	P1-2	T2-2

M67_FKDL210362718-1a	2020	1	China_Beijing	Asia	CAP	male	39	non-MUT	17	P1-1	T1-2
M69_FKDL210362720-1a	2020	1	China_Beijing	Asia	CAP	female	24	A2063G	14	P1-2	T2-2
M70_FKDL210362721-1a	2020	1	China_Beijing	Asia	CAP	male	9	A2063G	3	P1-1	T1-3R
M71_FKDL210362722-1a	2020	1	China_Beijing	Asia	CAP	male	5	A2063G	3	P1-1	T1-3R
M72_FKDL210362723-1a	2020	1	China_Beijing	Asia	CAP	female	5	A2063G	14	P1-2	T2-2
M73_FKDL210362724-1a	2020	1	China_Beijing	Asia	CAP	male	8	A2063G	3	P1-1	T1-3R
M74_FKDL210362725-1a	2020	1	China_Beijing	Asia	CAP	male	7	non-MUT	14	P1-2	T2-2
M79_FKDL210362730-1a	2020	1	China_Beijing	Asia	CAP	female	5	A2063G	3	P1-1	T1-3R
M80_FKDL210362731-1a	2019	12	China_Beijing	Asia	CAP	female	43	A2063G	3	P1-1	T1-3R
M81_FKDL210362732-1a	2020	1	China_Beijing	Asia	CAP	female	60	non-MUT	14	P1-2	T2-2
M82_FKDL210362733-1a	2020	1	China_Beijing	Asia	CAP	male	29	A2063G	14	P1-2	T2-2
M84_FKDL210362735-1a	2020	1	China_Beijing	Asia	SCAP	female	7	A2063G	3	P1-1	T1-3R
M85_FKDL210362736-1a	2020	1	China_Beijing	Asia	CAP	male	8	A2063G	3	P1-1	T1-3R
M86_FKDL210362737-1a	2020	1	China_Beijing	Asia	SCAP	female	8	A2063G	3	P1-1	T1-3R
M87_FKDL210362738-1a	2020	1	China_Beijing	Asia	CAP	male	11	A2063G	3	P1-1	T1-3R
M89_FKDL210362740-1a	2020	1	China_Beijing	Asia	SCAP	female	8	A2063G	3	P1-1	T1-3R
M90_FKDL210362741-1a	2020	1	China_Beijing	Asia	CAP	male	7	A2063G	3	P1-1	T1-3R
M91_FKDL210362742-1a	2020	1	China_Beijing	Asia	CAP	female	13	A2063G	3	P1-1	T1-3R
M92_FKDL210362743-1a	2020	1	China_Beijing	Asia	CAP	female	12	A2063G	3	P1-1	T1-3R
M93_FKDL210362744-1a	2020	1	China_Beijing	Asia	CAP	male	43	A2063G	3	P1-1	T1-3R
M95_FKDL210362746-1a	2020	1	China_Beijing	Asia	CAP	female	8	A2063G	3	P1-1	T1-3R
BJCDC-1	2021	7	China_Beijing	Asia	SCAP	female	8	A2063G	14	P1-2	T2-2
BJCDC-2	2021	7	China_Beijing	Asia	SCAP	male	7	A2063G	3	P1-1	T1-3R
BJCDC-3	2021	7	China_Beijing	Asia	CAP	female	7	A2063G	14	P1-2	T2-2
BJCDC-4	2021	9	China_Beijing	Asia	CAP	female	29	A2063G	3	P1-1	T1-3R
BJCDC-5	2021	9	China_Beijing	Asia	SCAP	male	4	A2063G	3	P1-1	T1-3R
BJCDC-6	2021	10	China_Beijing	Asia	CAP	female	27	A2063G	14	P1-2	T2-2
BJCDC-7	2021	10	China_Beijing	Asia	URTI	male	3	A2063G	14	P1-2	T2-2
BJCDC-8	2021	10	China_Beijing	Asia	URTI	female	9	A2063G	3	P1-1	T1-3R
BJCDC-9	2021	11	China_Beijing	Asia	CAP	male	9	A2063G	14	P1-2	T2-2
BJCDC-10	2021	11	China_Beijing	Asia	CAP	male	4	A2063G	14	P1-2	T2-2
BJCDC-11	2021	10	China_Beijing	Asia	CAP	male	4	A2063G	3	P1-1	T1-3R
BJCDC-12	2021	10	China_Beijing	Asia	CAP	male	8	A2063G	3	P1-1	T1-3R
BJCDC-13	2021	11	China_Beijing	Asia	CAP	male	65	non-MUT	14	P1-2	T2-2
BJCDC-14	2021	12	China_Beijing	Asia	CAP	male	25	A2063G	14	P1-2	T2-2
BJCDC-15	2021	11	China_Beijing	Asia	CAP	female	39	A2063G	14	P1-2	T2-2
BJCDC-16	2021	11	China_Beijing	Asia	CAP	male	42	A2063G	14	P1-2	T2-2
BJCDC-17	2021	12	China_Beijing	Asia	SCAP	female	7	A2063G	3	P1-1	T1-3R
BJCDC-18	2021	12	China_Beijing	Asia	CAP	female	2	A2063G	14	P1-2	T2-2

BJCDC-19	2021	12	China_Beijing	Asia	SCAP	female	8	A2063G	14	P1-2	T2-2
BJCDC-20	2021	12	China_Beijing	Asia	SCAP	female	7	A2063G	14	P1-2	T2-2
BJCDC-21	2022	2	China_Beijing	Asia	CAP	female	3	A2063G	3	P1-1	T1-3R
BJCDC-22	2022	2	China_Beijing	Asia	CAP	male	2	A2063G	3	P1-1	T1-3R
BJCDC-23	2022	2	China_Beijing	Asia	CAP	male	6	A2063G	14	P1-2	T2-2
BJCDC-24	2022	3	China_Beijing	Asia	SCAP	male	2	A2063G	3	P1-1	T1-3R
BJCDC-26	2022	4	China_Beijing	Asia	URTI	male	5	A2063G	3	P1-1	T1-3R
BJCDC-27	2022	6	China_Beijing	Asia	CAP	male	17	A2063G	14	P1-2	T2-2
BJCDC-28	2022	7	China_Beijing	Asia	CAP	male	4	A2063G	3	P1-1	T1-3R
BJCDC-29	2022	7	China_Beijing	Asia	SCAP	female	14	A2063G	14	P1-2	T2-2
BJCDC-30	2022	8	China_Beijing	Asia	CAP	male	4	A2063G	3	P1-1	T1-3R
BJCDC-31	2022	9	China_Beijing	Asia	CAP	female	3	A2063G	3	P1-1	T1-3R
BJCDC-32	2022	9	China_Beijing	Asia	CAP	male	32	A2063G	3	P1-1	T1-3R
BJCDC-33	2022	9	China_Beijing	Asia	SCAP	female	3	A2063G	14	P1-2	T2-2
BJCDC-34	2022	9	China_Beijing	Asia	CAP	male	6	A2063G	14	P1-2	T2-2
BJCDC-35	2022	9	China_Beijing	Asia	SCAP	male	4	A2063G	14	P1-2	T2-2
BJCDC-36	2022	10	China_Beijing	Asia	CAP	female	1	A2063G	3	P1-1	T1-3R
BJCDC-37	2022	10	China_Beijing	Asia	CAP	female	63	A2063G	3	P1-1	T1-3R
BJCDC-38	2022	10	China_Beijing	Asia	CAP	female	1	A2063G	3	P1-1	T1-3R
BJCDC-39	2022	10	China_Beijing	Asia	CAP	male	8	A2063G	14	P1-2	T2-2
BJCDC-40	2022	11	China_Beijing	Asia	CAP	female	5	A2063G	14	P1-2	T2-2
BJCDC-41	2023	1	China_Beijing	Asia	CAP	male	10	A2063G	14	P1-2	T2-2
BJCDC-42	2023	7	China_Beijing	Asia	SCAP	female	8	A2063G	3	P1-1	T1-3R
BJCDC-43	2023	7	China_Beijing	Asia	CAP	male	9	A2063G	3	P1-1	T1-3R
BJCDC-44	2023	7	China_Beijing	Asia	CAP	female	8	A2063G	3	P1-1	T1-3R
BJCDC-45	2023	7	China_Beijing	Asia	CAP	male	2	A2063G	14	P1-2	T2-2
BJCDC-46	2022	7	China_Beijing	Asia	SCAP	male	7	A2063G	14	P1-2	T2-2
BJCDC-47	2022	4	China_Beijing	Asia	SCAP	male	8	A2063G	14	P1-2	T2-2
BJCDC-48	2023	8	China_Beijing	Asia	other	male	8	A2063G	3	P1-1	T1-3R
BJCDC-49	2023	8	China_Beijing	Asia	CAP	male	6	A2063G	14	P1-2	T2-2
BJCDC-50	2023	8	China_Beijing	Asia	SCAP	female	42	A2063G	3	P1-1	T1-3R
BJCDC-51	2023	8	China_Beijing	Asia	CAP	female	40	A2063G	14	P1-2	T2-2
BJCDC-53	2023	8	China_Beijing	Asia	CAP	female	3	A2063G	3	P1-1	T1-3R
BJCDC-54	2023	8	China_Beijing	Asia	CAP	male	6	A2063G	3	P1-1	T1-3R
BJCDC-55	2023	8	China_Beijing	Asia	CAP	female	5	A2063G	14	P1-2	T2-2
BJCDC-56	2023	8	China_Beijing	Asia	CAP	female	8	A2063G	3	P1-1	T1-3R
BJCDC-57	2023	8	China_Beijing	Asia	CAP	male	4	A2063G	3	P1-1	T1-3R
BJCDC-58	2023	8	China_Beijing	Asia	CAP	male	2	A2063G	3	P1-1	T1-3R

BJCDC-59	2023	8	China_Beijing	Asia	CAP	female	7	A2063G	3	P1-1	T1-3R
BJCDC-60	2023	8	China_Beijing	Asia	CAP	female	9	A2063G	3	P1-1	T1-3R
BJCDC-61	2023	8	China_Beijing	Asia	CAP	female	12	A2063G	14	P1-2	T2-2
BJCDC-62	2023	8	China_Beijing	Asia	CAP	male	12	A2063G	14	P1-2	T2-2
BJCDC-63	2023	8	China_Beijing	Asia	CAP	male	6	A2063G	3	P1-1	T1-3R
BJCDC-64	2023	8	China_Beijing	Asia	CAP	male	9	A2063G	3	P1-1	T1-3R
BJCDC-65	2023	8	China_Beijing	Asia	URTI	male	29	A2063G	14	P1-2	T2-2
BJCDC-66	2023	8	China_Beijing	Asia	CAP	female	55	A2063G	3	P1-1	T1-3R
BJCDC-67	2023	8	China_Beijing	Asia	CAP	male	29	A2063G	14	P1-2	T2-2
BJCDC-68	2023	8	China_Beijing	Asia	CAP	female	6	A2063G	3	P1-1	T1-3R
BJCDC-69	2023	8	China_Beijing	Asia	CAP	male	6	A2063G	3	P1-1	T1-3R
BJCDC-70	2023	8	China_Beijing	Asia	CAP	female	10	A2063G	3	P1-1	T1-3R
BJCDC-71	2023	9	China_Beijing	Asia	CAP	male	8	A2063G	3	P1-1	T1-3R
BJCDC-72	2023	9	China_Beijing	Asia	other	female	6	A2063G	14	P1-2	T2-2
BJCDC-73	2023	9	China_Beijing	Asia	CAP	female	17	A2063G	14	P1-2	T2-2
BJCDC-74	2023	9	China_Beijing	Asia	CAP	male	76	A2063G	14	P1-2	T2-2
BJCDC-76	2023	9	China_Beijing	Asia	CAP	female	62	A2063G	3	P1-1	T1-3R
BJCDC-77	2023	9	China_Beijing	Asia	CAP	male	51	A2063G	14	P1-2	T2-2
BJCDC-78	2023	8	China_Beijing	Asia	CAP	male	15	A2063G	3	P1-1	T1-3R
BJCDC-79	2023	9	China_Beijing	Asia	CAP	female	5	A2063G	3	P1-1	T1-3R
BJCDC-80	2023	7	China_Beijing	Asia	CAP	female	37	A2063G	3	P1-1	T1-3R
mp-1_S1	2023	11	China_Beijing	Asia	CAP	male	10	A2063G	3	P1-1	T1-3R
mp-2_S2	2023	11	China_Beijing	Asia	CAP	female	5	A2063G	3	P1-1	T1-3R
mp-3_S3	2023	11	China_Beijing	Asia	CAP	female	10	A2063G	3	P1-1	T1-3R
mp-4_S4	2023	11	China_Beijing	Asia	CAP	male	11	A2063G	3	P1-1	T1-3R
mp-5_S5	2023	11	China_Beijing	Asia	CAP	male	8	A2063G	3	P1-1	T1-3R
mp-6_S6	2023	11	China_Beijing	Asia	CAP	male	6	A2063G	3	P1-1	T1-3R
mp-7_S7	2023	11	China_Beijing	Asia	CAP	male	6	A2063G	14	P1-2	T2-2
mp-8_S8	2023	11	China_Beijing	Asia	CAP	female	7	A2063G	3	P1-1	T1-3R

Supplementary Table 2. Metadata of SRA raw data and genomes from public database.

non-MUT: non-mutation.

ID	Year	Region	Continent	23S rRNA mutations	ST	P1 subtype	Phylogenetic tree subclade
DRR040043	2008	Japan	Asia	A2063G	3	P1-1	T1-3

DRR040044	2008	Japan	Asia	A2063G	3	P1-1	T1-3
DRR040045	2008	Japan	Asia	non-MUT	19	P1-1	T1-3
DRR040046	2008	Japan	Asia	non-MUT	3	P1-1	T1-3
DRR040047	2009	Japan	Asia	A2063G	3	P1-1	T1-3
DRR040048	2010	Japan	Asia	A2063G	3	P1-1	T1-3
DRR040049	2011	Japan	Asia	non-MUT	14	P1-2	T2-2
DRR040050	2011	Japan	Asia	non-MUT	19	P1-1	T1-3
DRR040051	2011	Japan	Asia	A2063G	3	P1-1	T1-3
DRR040052	2011	Japan	Asia	A2063G	3	P1-1	T1-3
DRR040053	2011	Japan	Asia	non-MUT	3	P1-1	T1-3
DRR040054	2012	Japan	Asia	A2063G	3	P1-1	T1-3R
DRR040055	1976	Japan	Asia	non-MUT	3	P1-1	T1-3
DRR040056	1984	Japan	Asia	non-MUT	3	P1-1	T1-3
DRR040057	1986	Japan	Asia	non-MUT	3	P1-1	T1-3
DRR040058	1987	Japan	Asia	non-MUT	3	P1-1	T1-3
ERR949844	N. D.	N. D.	N. D.	non-MUT	2	P1-2	T2-1
ERR974260	1981	UK	Europe	non-MUT	2	P1-2	T2-1
ERR974261	1981	UK	Europe	non-MUT	2	P1-2	T2-1
ERR974262	1986	UK	Europe	non-MUT	1	P1-1	T1-1
ERR974263	1981	UK	Europe	non-MUT	2	P1-2	T2-1
ERR974264	1978	UK	Europe	non-MUT	2	P1-2	T2-1
ERR974265	1982	UK	Europe	non-MUT	3	P1-1	T1-3
ERR974266	1967	UK	Europe	non-MUT	3	P1-1	T1-3
ERR974267	1996	UK	Europe	non-MUT	3	P1-1	T1-3
ERR974268	1968	UK	Europe	non-MUT	5	P1-1	T1-3
ERR974269	1983	UK	Europe	non-MUT	9	P1-1	T1-3
ERR974270	1983	UK	Europe	non-MUT	2	P1-2	T2-1
ERR974271	1983	UK	Europe	non-MUT	6	P1-2	T2-1
ERR974272	1982	UK	Europe	non-MUT	2	P1-2	T2-1
ERR974273	1983	UK	Europe	non-MUT	3	P1-1	T1-3
ERR974274	1982	UK	Europe	non-MUT	2	P1-2	T2-1
ERR974275	1983	UK	Europe	non-MUT	2	P1-2	T2-1
ERR974276	1982	UK	Europe	non-MUT	3	P1-1	T1-3
ERR974277	1982	UK	Europe	non-MUT	3	P1-1	T1-3
ERR974278	1982	UK	Europe	non-MUT	3	P1-1	T1-3
ERR974279	1983	UK	Europe	non-MUT	2	P1-2	T2-1
ERR974280	1983	UK	Europe	non-MUT	8	P1-2	T2-1
ERR974281	1981	UK	Europe	non-MUT	2	P1-2	T2-1

ERR974282	1981	UK	Europe	non-MUT	2	P1-2	T2-1
ERR974283	1983	UK	Europe	non-MUT	3	P1-1	T1-3
ERR974284	1979	UK	Europe	non-MUT	7	P1-2	T2-1
ERR974285	1981	UK	Europe	non-MUT	2	P1-2	T2-1
ERR974286	1982	UK	Europe	non-MUT	2	P1-2	T2-1
ERR974287	1976	UK	Europe	non-MUT	2	P1-2	T2-1
ERR974288	1982	UK	Europe	non-MUT	3	P1-1	T1-3
ERR974289	1982	UK	Europe	non-MUT	2	P1-2	T2-1
ERR974290	1982	UK	Europe	non-MUT	2	P1-2	T2-1
ERR974291	1983	UK	Europe	non-MUT	2	P1-2	T2-1
ERR974292	1982	UK	Europe	non-MUT	2	P1-2	T2-1
ERR974293	1983	UK	Europe	non-MUT	2	P1-2	T2-1
ERR974294	1982	UK	Europe	non-MUT	3	P1-1	T1-3
GCA_000143945.1	N. D.	N. D.	N. D.	non-MUT	2	P1-2	T2-1
GCA_000283755.1	N. D.	N. D.	N. D.	non-MUT	2	P1-2	T2-2
GCA_000319655.2	1980	USA	North America	non-MUT	2	P1-2	T2-1
GCA_000319675.2	N. D.	USA	North America	non-MUT	3	P1-1	T1-3
GCA_000331085.2	N. D.	USA	North America	non-MUT	1	P1-1	T1-1
GCA_000387745.2	1994	USA	North America	non-MUT	7	P1-2	T2-1
GCA_000733995.1	2005	China_Beijing	Asia	A2063G	3	P1-1	T1-3R
GCA_001272715.1	1999	USA	North America	non-MUT	2	P1-2	T2-1
GCA_001272735.1	2006	USA	North America	non-MUT	20	P1-1	T1-3
GCA_001272755.1	2009	USA	North America	A2063G	20	P1-1	T1-3
GCA_001272775.1	2009	USA	North America	non-MUT	20	P1-1	T1-3
GCA_001272795.1	1985	China	Asia	non-MUT	3	P1-1	T1-3
GCA_001272815.1	1985	China	Asia	non-MUT	3	P1-1	T1-3
GCA_001272835.1	N. D.	USA	North America	non-MUT	2	P1-2	T2-1
GCA_001272855.1	1981	UK	Europe	non-MUT	2	P1-2	T2-1
GCA_001272875.1	1982	UK	Europe	non-MUT	7	P1-2	T2-1
GCA_001272895.1	1982	UK	Europe	non-MUT	2	P1-2	T2-1
GCA_001272915.1	N. D.	USA	North America	non-MUT	2	P1-2	T2-1
GCA_001296485.1	1999	France	Europe	non-MUT	20	P1-1	T1-3
GCA_001296505.1	1996	France	Europe	non-MUT	3	P1-1	T1-3
GCA_001296515.1	2001	France	Europe	non-MUT	7	P1-2	T2-1
GCA_001296525.1	1999	Spain	Europe	non-MUT	2	P1-2	T2-1
GCA_001296565.1	2005	France	Europe	non-MUT	14	P1-2	T2-2
GCA_001296585.1	2005	France	Europe	non-MUT	3	P1-1	T1-3
GCA_001296605.1	2000/2003	Japan	Asia	non-MUT	7	P1-2	T2-1

GCA_001296615.1	2006	Tunisia	North America	non-MUT	1	P1-1	T1-1
GCA_001296625.1	2008	France	Europe	non-MUT	2	P1-2	T2-1
GCA_001296665.1	1993	Germany	Europe	non-MUT	3	P1-1	T1-3
GCA_001296685.1	1991	Germany	Europe	non-MUT	2	P1-2	T2-2
GCA_001296705.1	2011	France	Europe	non-MUT	20	P1-1	T1-3
GCA_001296725.1	2011	France	Europe	non-MUT	3	P1-1	T1-3
GCA_001296735.1	2011	France	Europe	A2063G	3	P1-1	T1-3
GCA_001296765.1	2011	France	Europe	non-MUT	14	P1-2	T2-2
GCA_001296785.1	2011	France	Europe	non-MUT	2	P1-2	T2-1
GCA_001296805.1	1967	Denmark	Europe	non-MUT	2	P1-2	T2-1
GCA_001296815.1	2005	France	Europe	non-MUT	3	P1-1	T1-3
GCA_001296825.1	2000/2003	Japan	Asia	A2063C	7	P1-2	T2-1
GCA_001296855.1	2008	Tunisia	North America	non-MUT	1	P1-1	T1-1
GCA_001296885.1	2011	France	Europe	non-MUT	3	P1-1	T1-3
GCA_001296895.1	2011	France	Europe	non-MUT	3	P1-1	T1-3
GCA_001296905.1	1970/1979	France	Europe	non-MUT	3	P1-1	T1-3
GCA_001455605.1	2010	N. D.	N. D.	A2063G	3	P1-1	T1-3R
GCA_001455625.1	2012	N. D.	N. D.	A2063G	3	P1-1	T1-3R
GCA_001455635.1	2010	N. D.	N. D.	A2063G	3	P1-1	T1-3R
GCA_001455675.1	2012	N. D.	N. D.	A2063G	3	P1-1	T1-3R
GCA_001455685.1	2012	N. D.	N. D.	A2063G	3	P1-1	T1-3R
GCA_001455695.1	2012	N. D.	N. D.	A2063G	3	P1-1	T1-3R
GCA_001455735.1	2012	N. D.	N. D.	A2063G	3	P1-1	T1-3R
GCA_001455745.1	2012	N. D.	N. D.	A2063G	3	P1-1	T1-3R
GCA_001455775.1	2012	N. D.	N. D.	A2063G	3	P1-1	T1-3R
GCA_001455795.1	2012	N. D.	N. D.	A2063G	30	P1-1	T1-3
GCA_001509195.1	2012	China_Beijing	Asia	A2063G	3	P1-1	T1-3R
GCA_001558175.1	2012	China_Beijing	Asia	A2063G	3	P1-1	T1-3R
GCA_001901705.1	1954	USA	North America	non-MUT	2	P1-2	T2-1
GCA_002090215.1	2016	China_Beijing	Asia	A2063G	1	P1-1	T1-1
GCA_002090235.1	2016	China_Beijing	Asia	non-MUT	1	P1-1	T1-1
GCA_002090275.1	2015	China_Beijing	Asia	A2063G	1	P1-1	T1-1
GCA_002090295.1	2015	China_Beijing	Asia	A2063G	1	P1-1	T1-1
GCA_002090315.1	2016	China_Beijing	Asia	A2063G	1	P1-1	T1-1
GCA_002095995.1	2016	China_Beijing	Asia	A2063G	1	P1-1	T1-1
GCA_002096015.1	2016	China_Beijing	Asia	A2063G	1	P1-1	T1-1
GCA_002096035.1	2016	China_Beijing	Asia	A2063G	1	P1-1	T1-1

GCA_002127985.1	1988	Denmark	Europe	A2063G	19	P1-1	T1-3
GCA_002128005.1	2009	Egypt	Africa	non-MUT	14	P1-2	T2-2
GCA_002128025.1	1965	USA	North America	non-MUT	1	P1-1	T1-1
GCA_002128045.1	2012	USA	North America	non-MUT	3	P1-1	T1-3
GCA_002128065.1	2010	Egypt	Africa	non-MUT	20	P1-1	T1-3
GCA_002128085.1	2007	USA	North America	non-MUT	2	P1-2	T2-1
GCA_002128105.1	2000	USA	North America	non-MUT	2	P1-2	T2-1
GCA_002128125.1	2014	USA	North America	non-MUT	14	P1-2	T2-2
GCA_002128145.1	2012	USA	North America	non-MUT	20	P1-1	T1-3
GCA_002128165.1	2013	USA	North America	non-MUT	2	P1-2	T2-1
GCA_002128185.1	2010	Kenya	Africa	non-MUT	16	P1-2	T2-1
GCA_002128205.1	1999	USA	North America	A2063G	7	P1-2	T2-1
GCA_002128235.1	1999	USA	North America	non-MUT	2	P1-2	T2-1
GCA_002128265.1	1995	USA	North America	non-MUT	15	P1-2	T2-2
GCA_002128285.1	2012	USA	North America	non-MUT	2	P1-2	T2-1
GCA_002147855.1	1968	USA	North America	non-MUT	1	P1-1	T1-1
GCA_002355695.1	2012	Japan	Asia	non-MUT	2	P1-2	T2-1
GCA_002355715.1	2012	Japan	Asia	non-MUT	14	P1-2	T2-2
GCA_002563345.1	2013	USA	North America	non-MUT	3	P1-1	T1-3
GCA_002563355.1	N. D.	Denmark	Europe	non-MUT	2	P1-2	T2-1
GCA_002563365.1	1999	USA	North America	non-MUT	7	P1-2	T2-1
GCA_002563415.1	1998	Kenya	Africa	non-MUT	3	P1-1	T1-3
GCA_002563435.1	2010	Kenya	Africa	non-MUT	3	P1-1	T1-3
GCA_002563495.1	1991	USA	North America	non-MUT	1	P1-1	T1-1
GCA_002563515.1	2010	Guatemala	North America	non-MUT	3	P1-1	T1-3
GCA_002563545.1	1954	USA	North America	non-MUT	2	P1-2	T2-1
GCA_009809995.1	1976	Japan	Asia	non-MUT	7	P1-2	T2-1
GCA_009810015.1	1980	Japan	Asia	non-MUT	7	P1-2	T2-1
GCA_009810035.1	1983	Japan	Asia	non-MUT	7	P1-2	T2-1
GCA_009810055.1	1984	Japan	Asia	non-MUT	7	P1-2	T2-1
GCA_009810095.1	1989	Japan	Asia	non-MUT	3	P1-1	T1-3
GCA_009810115.1	1992	Japan	Asia	non-MUT	3	P1-1	T1-3
GCA_009810135.1	1992	Japan	Asia	non-MUT	7	P1-2	T2-1
GCA_009810155.1	2011	Japan	Asia	non-MUT	14	P1-2	T2-2
GCA_009810175.1	2013	Japan	Asia	non-MUT	14	P1-2	T2-2
GCA_009810195.1	2013	Japan	Asia	non-MUT	16	P1-2	T2-1
GCA_009810235.1	2011	Japan	Asia	A2063T	3	P1-1	T1-3
GCA_009810255.1	2011	Japan	Asia	non-MUT	14	P1-2	T2-2
GCA_009810275.1	2011	Japan	Asia	non-MUT	14	P1-2	T2-2

GCA_009810295.1	2008	Japan	Asia	non-MUT	3	P1-1	T1-3
GCA_009810315.1	1979	Japan	Asia	non-MUT	7	P1-2	T2-1
GCA_009810335.1	1980	Japan	Asia	non-MUT	7	P1-2	T2-1
GCA_009810355.1	1983	Japan	Asia	non-MUT	3	P1-1	T1-3
GCA_009810375.1	1985	Japan	Asia	non-MUT	7	P1-2	T2-1
GCA_009810395.1	1985	Japan	Asia	non-MUT	3	P1-1	T1-3
GCA_009810415.1	1987	Japan	Asia	non-MUT	7	P1-2	T2-1
GCA_009810435.1	1988	Japan	Asia	non-MUT	3	P1-1	T1-3
GCA_009810455.1	1988	Japan	Asia	non-MUT	3	P1-1	T1-3
GCA_009810475.1	1990	Japan	Asia	non-MUT	3	P1-1	T1-3R
GCA_009810495.1	1991	Japan	Asia	non-MUT	2	P1-2	T2-2
GCA_009810515.1	1991	Japan	Asia	non-MUT	2	P1-2	T2-2
GCA_009810535.1	1993	Japan	Asia	non-MUT	2	P1-2	T2-2
GCA_009810555.1	1994	Japan	Asia	non-MUT	7	P1-2	T2-1
GCA_009810575.1	2013	Japan	Asia	A2063G	3	P1-1	T1-3R
GCA_009810595.1	2008	Japan	Asia	non-MUT	3	P1-1	T1-3
GCA_009810615.1	2009	Japan	Asia	non-MUT	14	P1-2	T2-2
GCA_009810635.1	2010	Japan	Asia	non-MUT	14	P1-2	T2-2
GCA_009810655.1	2011	Japan	Asia	non-MUT	14	P1-2	T2-2
GCA_009810675.1	2011	Japan	Asia	non-MUT	14	P1-2	T2-2
GCA_009810695.1	2012	Japan	Asia	A2063T	3	P1-1	T1-3
GCA_009810715.1	2012	Japan	Asia	non-MUT	14	P1-2	T2-2
GCA_009810735.1	2012	Japan	Asia	A2063G	3	P1-1	T1-3R
GCA_009810755.1	2012	Japan	Asia	A2063T	3	P1-1	T1-3
GCA_009810775.1	1985	Japan	Asia	non-MUT	3	P1-1	T1-3
GCA_009810795.1	2016	Japan	Asia	A2063G	3	P1-1	T1-3R
GCA_009810815.1	2016	Japan	Asia	A2063G	3	P1-1	T1-3R
GCA_009810835.1	2016	Japan	Asia	A2063G	3	P1-1	T1-3R
GCA_009810855.1	2011	Japan	Asia	non-MUT	14	P1-2	T2-2
GCA_009810875.1	2011	Japan	Asia	non-MUT	14	P1-2	T2-2
GCA_009810895.1	2011	Japan	Asia	non-MUT	7	P1-2	T2-1
GCA_009810915.1	2011	Japan	Asia	A2063G	3	P1-1	T1-3
GCA_009810935.1	2011	Japan	Asia	non-MUT	14	P1-2	T2-2
GCA_009810955.1	2011	Japan	Asia	A2063G	3	P1-1	T1-3R
GCA_009810975.1	2011	Japan	Asia	non-MUT	3	P1-1	T1-3
GCA_009810995.1	2011	Japan	Asia	A2063G	14	P1-2	T2-2
GCA_009811015.1	2011	Japan	Asia	A2063G	3	P1-1	T1-3R
GCA_009811035.1	2011	Japan	Asia	non-MUT	14	P1-2	T2-2

GCA_009811055.1	2011	Japan	Asia	A2063G	3	P1-1	T1-3R
GCA_009811075.1	2011	Japan	Asia	non-MUT	14	P1-2	T2-2
GCA_009811095.1	2011	Japan	Asia	A2063G	3	P1-1	T1-3R
GCA_009811115.1	2011	Japan	Asia	non-MUT	14	P1-2	T2-2
GCA_009811135.1	2011	Japan	Asia	A2063G	30	P1-1	T1-3
GCA_009811155.1	2011	Japan	Asia	non-MUT	7	P1-2	T2-1
GCA_009811175.1	2011	Japan	Asia	A2063G	14	P1-2	T2-2
GCA_009811195.1	2011	Japan	Asia	non-MUT	14	P1-2	T2-2
GCA_009811215.1	2011	Japan	Asia	non-MUT	7	P1-2	T2-1
GCA_009811235.1	2011	Japan	Asia	A2063G	7	P1-2	T2-1
GCA_009811255.1	2011	Japan	Asia	non-MUT	3	P1-1	T1-3
GCA_009811275.1	2011	Japan	Asia	A2063G	3	P1-1	T1-3R
GCA_009811295.1	2011	Japan	Asia	non-MUT	7	P1-2	T2-1
GCA_009939745.1	2016	South_Korea	Asia	non-MUT	14	P1-2	T2-2
GCA_009939765.1	2016	South_Korea	Asia	A2063G	3	P1-1	T1-3R
GCA_009939785.1	2016	South_Korea	Asia	A2063G	3	P1-1	T1-3R
GCA_009939805.1	2016	South_Korea	Asia	A2063G	3	P1-1	T1-3R
GCA_009939825.1	2016	South_Korea	Asia	A2063G	3	P1-1	T1-3R
GCA_009939845.1	2016	South_Korea	Asia	A2063G	3	P1-1	T1-3R
GCA_009939975.1	2016	South_Korea	Asia	A2063G	3	P1-1	T1-3R
GCA_009940325.1	2015	South_Korea	Asia	A2063G	3	P1-1	T1-3R
GCA_009940965.1	2015	South_Korea	Asia	A2063G	3	P1-1	T1-3R
GCA_009941325.1	2015	South_Korea	Asia	A2063G	3	P1-1	T1-3R
GCA_009941705.1	2015	South_Korea	Asia	A2063G	3	P1-1	T1-3R
GCA_009942155.1	2014	South_Korea	Asia	non-MUT	14	P1-2	T2-2
GCA_009942395.1	2012	South_Korea	Asia	A2063G	3	P1-1	T1-3R
GCA_009942655.1	2012	South_Korea	Asia	A2063G	3	P1-1	T1-3R
GCA_009942915.1	2011	South_Korea	Asia	non-MUT	33	P1-2	T2-2
GCA_009943205.1	2011	South_Korea	Asia	non-MUT	3	P1-1	T1-3
GCA_009943505.1	2011	South_Korea	Asia	A2063G	14	P1-2	T2-2
GCA_009943805.1	2011	South_Korea	Asia	A2063G	3	P1-1	T1-3R
GCA_009944075.1	2011	South_Korea	Asia	non-MUT	3	P1-1	T1-3
GCA_009944335.1	2011	South_Korea	Asia	A2063G	3	P1-1	T1-3R
GCA_009944725.1	2011	South_Korea	Asia	non-MUT	14	P1-2	T2-2
GCA_009945165.1	2011	South_Korea	Asia	non-MUT	17	P1-1	T1-2
GCA_009945535.1	2011	South_Korea	Asia	non-MUT	3	P1-1	T1-3
GCA_009945865.1	2010	South_Korea	Asia	non-MUT	14	P1-2	T2-2
GCA_009946285.1	2010	South_Korea	Asia	non-MUT	3	P1-1	T1-3
GCA_009946845.1	2010	South_Korea	Asia	A2063G	3	P1-1	T1-3R

GCA_009947205.1	2010	South_Korea	Asia	non-MUT	1	P1-1	T1-1
GCA_009947575.1	2010	South_Korea	Asia	non-MUT	17	P1-1	T1-2
GCA_009947985.1	2010	South_Korea	Asia	A2063G	3	P1-1	T1-3R
GCA_009948395.1	2010	South_Korea	Asia	non-MUT	1	P1-1	T1-1
GCA_030159355.1	1980	Japan	Asia	non-MUT	8	P1-2	T2-1
GCA_030159375.1	2020	Japan	Asia	non-MUT	17	P1-1	T1-2
GCA_030159395.1	2020	Japan	Asia	A2063G	2	P1-2	T2-1
GCA_030159415.1	2020	Japan	Asia	non-MUT	7	P1-2	T2-1
GCA_030159435.1	2020	Japan	Asia	non-MUT	17	P1-1	T1-2
GCA_030159455.1	2020	Japan	Asia	A2063G	3	P1-1	T1-3R
GCA_030923165.1	2019	China_Taiwan	Asia	A2063G	3	P1-1	T1-3R
GCA_030923175.1	2019	China_Taiwan	Asia	non-MUT	14	P1-2	T2-2
GCA_030923185.1	2019	China_Taiwan	Asia	non-MUT	17	P1-1	T1-2
GCA_030923225.1	2019	China_Taiwan	Asia	A2063G	3	P1-1	T1-3R
GCA_030923245.1	2019	China_Taiwan	Asia	A2063T	17	P1-1	T1-2
GCA_030923285.1	2019	China_Taiwan	Asia	A2063G	3	P1-1	T1-3R
GCA_030923305.1	2019	China_Taiwan	Asia	A2063G	3	P1-1	T1-3R
GCA_030923315.1	2019	China_Taiwan	Asia	A2063G	17	P1-1	T1-2
GCA_030923325.1	2019	China_Taiwan	Asia	A2063G	3	P1-1	T1-3R
GCA_030923335.1	2019	China_Taiwan	Asia	non-MUT	17	P1-1	T1-2
GCA_030923395.1	2019	China_Taiwan	Asia	A2063G	3	P1-1	T1-3R
GCA_030923415.1	2019	China_Taiwan	Asia	A2063G	3	P1-1	T1-3R
GCA_030923435.1	2018	China_Taiwan	Asia	non-MUT	17	P1-1	T1-2
GCA_030923455.1	2018	China_Taiwan	Asia	non-MUT	14	P1-2	T2-2
GCA_030923475.1	2018	China_Taiwan	Asia	non-MUT	17	P1-1	T1-2
GCA_030923495.1	2018	China_Taiwan	Asia	A2063G	3	P1-1	T1-3R
GCA_030923515.1	2018	China_Taiwan	Asia	A2063G	3	P1-1	T1-3R
GCA_030923535.1	2019	China_Taiwan	Asia	A2063G	3	P1-1	T1-3R
GCA_030923555.1	2019	China_Taiwan	Asia	A2063G	3	P1-1	T1-3R
GCA_030923575.1	2019	China_Taiwan	Asia	A2063G	3	P1-1	T1-3R
GCA_030923585.1	2019	China_Taiwan	Asia	non-MUT	17	P1-1	T1-2
GCA_030923675.1	2019	China_Taiwan	Asia	A2063T	17	P1-1	T1-2
GCA_030923695.1	2019	China_Taiwan	Asia	A2063G	3	P1-1	T1-3R
GCA_030923755.1	2019	China_Taiwan	Asia	A2063G	3	P1-1	T1-3R
GCA_030923795.1	2019	China_Taiwan	Asia	A2063G	3	P1-1	T1-3R
GCA_030923825.1	2019	China_Taiwan	Asia	A2063G	3	P1-1	T1-3R
GCA_030923835.1	2019	China_Taiwan	Asia	A2063G	3	P1-1	T1-3R
GCA_030923875.1	2019	China_Taiwan	Asia	A2063G	3	P1-1	T1-3R

GCA_030923885.1	2019	China_Taiwan	Asia	A2063T	17	P1-1	T1-2
GCA_030924015.1	2019	China_Taiwan	Asia	A2063G	3	P1-1	T1-3R
GCA_030924025.1	2019	China_Taiwan	Asia	A2063G	3	P1-1	T1-3R
GCA_030924065.1	2019	China_Taiwan	Asia	A2063G	3	P1-1	T1-3R
GCA_030924095.1	2019	China_Taiwan	Asia	A2063T	17	P1-1	T1-2
GCA_030924105.1	2019	China_Taiwan	Asia	non-MUT	17	P1-1	T1-2
GCA_030924135.1	2019	China_Taiwan	Asia	A2063G	3	P1-1	T1-3R
GCA_030924155.1	2020	China_Taiwan	Asia	A2063G	3	P1-1	T1-3R
GCA_030924175.1	2019	China_Taiwan	Asia	A2063G	3	P1-1	T1-3R
GCA_030924195.1	2019	China_Taiwan	Asia	A2063G	3	P1-1	T1-3R
GCA_030924205.1	2019	China_Taiwan	Asia	A2063T	17	P1-1	T1-2
GCA_030924235.1	2020	China_Taiwan	Asia	A2063T	17	P1-1	T1-2
GCA_030924255.1	2020	China_Taiwan	Asia	A2063G	3	P1-1	T1-3R
GCA_030924265.1	2020	China_Taiwan	Asia	A2063G	3	P1-1	T1-3R
GCA_030924275.1	2020	China_Taiwan	Asia	A2063T	17	P1-1	T1-2
GCA_030924315.1	2020	China_Taiwan	Asia	A2063G	14	P1-2	T2-2
GCA_030924335.1	2019	China_Taiwan	Asia	A2063G	3	P1-1	T1-3R
GCA_030924355.1	2019	China_Taiwan	Asia	A2063G	3	P1-1	T1-3R
GCA_030924375.1	2019	China_Taiwan	Asia	A2063G	17	P1-1	T1-2
GCA_030924395.1	2019	China_Taiwan	Asia	A2063G	3	P1-1	T1-3R
GCA_030924405.1	2019	China_Taiwan	Asia	A2063G	3	P1-1	T1-3R
GCA_030924435.1	2019	China_Taiwan	Asia	A2063T	17	P1-1	T1-2
GCA_030924455.1	2019	China_Taiwan	Asia	A2063G	3	P1-1	T1-3R
GCA_030924475.1	2019	China_Taiwan	Asia	A2063G	3	P1-1	T1-3R
GCA_030924495.1	2019	China_Taiwan	Asia	A2063G	17	P1-1	T1-2
GCA_030924505.1	2019	China_Taiwan	Asia	A2063G	3	P1-1	T1-3R
GCA_030924535.1	2019	China_Taiwan	Asia	A2063G	17	P1-1	T1-2
GCA_030924545.1	2019	China_Taiwan	Asia	non-MUT	14	P1-2	T2-2
GCA_030924575.1	2019	China_Taiwan	Asia	A2063G	17	P1-1	T1-2
GCA_030924595.1	2019	China_Taiwan	Asia	A2063G	3	P1-1	T1-3R
GCA_030924615.1	2019	China_Taiwan	Asia	A2063G	3	P1-1	T1-3R
GCA_030924635.1	2019	China_Taiwan	Asia	A2063G	3	P1-1	T1-3R
GCA_030924655.1	2019	China_Taiwan	Asia	A2063G	14	P1-2	T2-2
GCA_030924675.1	2019	China_Taiwan	Asia	A2063G	3	P1-1	T1-3R
GCA_030924695.1	2019	China_Taiwan	Asia	A2063G	17	P1-1	T1-2
GCA_030924705.1	2019	China_Taiwan	Asia	A2063T	17	P1-1	T1-2
GCA_030924715.1	2019	China_Taiwan	Asia	A2063G	3	P1-1	T1-3R
GCA_030924755.1	2019	China_Taiwan	Asia	A2063G	3	P1-1	T1-3R
GCA_030924775.1	2019	China_Taiwan	Asia	A2063G	3	P1-1	T1-3R

GCA_030924795.1	2019	China_Taiwan	Asia	A2063G	3	P1-1	T1-3R
GCA_030924815.1	2019	China_Taiwan	Asia	A2063G	3	P1-1	T1-3R
GCA_030924825.1	2019	China_Taiwan	Asia	A2063G	3	P1-1	T1-3R
GCA_030924835.1	2019	China_Taiwan	Asia	non-MUT	14	P1-2	T2-2
GCA_030924875.1	2019	China_Taiwan	Asia	A2063G	3	P1-1	T1-3R
GCA_030924895.1	2019	China_Taiwan	Asia	A2063G	3	P1-1	T1-3R
GCA_030924915.1	2019	China_Taiwan	Asia	non-MUT	17	P1-1	T1-2
GCA_030924925.1	2019	China_Taiwan	Asia	non-MUT	2	P1-2	T2-1
GCA_030924935.1	2019	China_Taiwan	Asia	A2063G	3	P1-1	T1-3R
GCA_030924945.1	2019	China_Taiwan	Asia	A2063G	17	P1-1	T1-2
GCA_030924995.1	2019	China_Taiwan	Asia	A2063G	3	P1-1	T1-3R
GCA_030925015.1	2019	China_Taiwan	Asia	A2063T	17	P1-1	T1-2
GCA_030925035.1	2018	China_Taiwan	Asia	A2063G	3	P1-1	T1-3R
GCA_030925055.1	2018	China_Taiwan	Asia	A2063G	3	P1-1	T1-3R
GCA_030925075.1	2018	China_Taiwan	Asia	non-MUT	14	P1-2	T2-2
GCA_030925085.1	2018	China_Taiwan	Asia	A2063G	17	P1-1	T1-2
GCA_030925115.1	2018	China_Taiwan	Asia	A2063G	17	P1-1	T1-2
GCA_030925135.1	2018	China_Taiwan	Asia	A2063G	17	P1-1	T1-2
GCA_030925155.1	2018	China_Taiwan	Asia	A2063G	17	P1-1	T1-2
GCA_030925165.1	2018	China_Taiwan	Asia	A2063G	17	P1-1	T1-2
GCA_030925175.1	2018	China_Taiwan	Asia	A2063G	3	P1-1	T1-3R
GCA_030925215.1	2020	China_Taiwan	Asia	A2063G	3	P1-1	T1-3R
GCA_030925235.1	2019	China_Taiwan	Asia	A2063G	17	P1-1	T1-2
GCA_030925245.1	2019	China_Taiwan	Asia	A2063G	3	P1-1	T1-3R
GCA_030925255.1	2020	China_Taiwan	Asia	A2063G	17	P1-1	T1-2
GCA_030925295.1	2019	China_Taiwan	Asia	A2063G	3	P1-1	T1-3R
GCA_030925315.1	2019	China_Taiwan	Asia	A2063G	3	P1-1	T1-3R
GCA_030925335.1	2019	China_Taiwan	Asia	A2063G	3	P1-1	T1-3R
GCA_030925355.1	2019	China_Taiwan	Asia	A2063G	3	P1-1	T1-3R
GCA_030925375.1	2019	China_Taiwan	Asia	A2063G	3	P1-1	T1-3R
GCA_030925385.1	2019	China_Taiwan	Asia	A2063G	3	P1-1	T1-3R
GCA_030928045.1	2019	China_Taiwan	Asia	A2063G	3	P1-1	T1-3R
GCA_900660465.1	N. D.	N. D.	N. D.	non-MUT	2	P1-2	T2-1
GCA_910574535.1	N. D.	N. D.	N. D.	non-MUT	1	P1-1	T1-1
SRR11193106	2019	China_Taiwan	Asia	non-MUT	17	P1-1	T1-2
SRR11193107	2019	China_Taiwan	Asia	A2063T	17	P1-1	T1-2
SRR11193108	2019	China_Taiwan	Asia	A2063G	3	P1-1	T1-3R
SRR11193109	2019	China_Taiwan	Asia	A2063G	3	P1-1	T1-3R

SRR11193110	2019	China_Taiwan	Asia	A2063G	3	P1-1	T1-3R
SRR11193111	2019	China_Taiwan	Asia	A2063G	17	P1-1	T1-2
SRR11193112	2019	China_Taiwan	Asia	A2063G	3	P1-1	T1-3R
SRR11193113	2018	China_Taiwan	Asia	non-MUT	17	P1-1	T1-2
SRR11193114	2018	China_Taiwan	Asia	non-MUT	14	P1-2	T2-2
SRR11193115	2019	China_Taiwan	Asia	A2063T	17	P1-1	T1-2
SRR11193116	2019	China_Taiwan	Asia	A2063G	3	P1-1	T1-3R
SRR11193117	2019	China_Taiwan	Asia	A2063G	3	P1-1	T1-3R
SRR11193118	2019	China_Taiwan	Asia	A2063G	3	P1-1	T1-3R
SRR11193119	2019	China_Taiwan	Asia	A2063G	3	P1-1	T1-3R
SRR11193120	2019	China_Taiwan	Asia	A2063G	3	P1-1	T1-3R
SRR11193121	2019	China_Taiwan	Asia	A2063G	3	P1-1	T1-3R
SRR11193122	2019	China_Taiwan	Asia	A2063G	3	P1-1	T1-3R
SRR11193123	2019	China_Taiwan	Asia	A2063T	17	P1-1	T1-2
SRR11193124	2019	China_Taiwan	Asia	A2063G	3	P1-1	T1-3R
SRR11193125	2019	China_Taiwan	Asia	A2063G	3	P1-1	T1-3R
SRR11193126	2019	China_Taiwan	Asia	A2063G	14	P1-2	T2-2
SRR11193127	2019	China_Taiwan	Asia	A2063G	17	P1-1	T1-2
SRR11193128	2019	China_Taiwan	Asia	A2063G	3	P1-1	T1-3R
SRR11193129	2019	China_Taiwan	Asia	A2063G	3	P1-1	T1-3R
SRR11193130	2019	China_Taiwan	Asia	A2063G	3	P1-1	T1-3R
SRR11193131	2019	China_Taiwan	Asia	A2063G	17	P1-1	T1-2
SRR11193132	2018	China_Taiwan	Asia	A2063G	3	P1-1	T1-3R
SRR11193133	2018	China_Taiwan	Asia	A2063G	17	P1-1	T1-2
SRR11193134	2019	China_Taiwan	Asia	A2063G	3	P1-1	T1-3R
SRR11193135	2019	China_Taiwan	Asia	A2063G	3	P1-1	T1-3R
SRR11193136	2019	China_Taiwan	Asia	A2063G	3	P1-1	T1-3R
SRR11193137	2019	China_Taiwan	Asia	A2063G	3	P1-1	T1-3R
SRR12190402	2018	China_Taiwan	Asia	A2063G	3	P1-1	T1-3R
SRR12190403	2019	China_Taiwan	Asia	A2063G	3	P1-1	T1-3R
SRR12190404	2019	China_Taiwan	Asia	A2063G	3	P1-1	T1-3R
SRR12190405	2019	China_Taiwan	Asia	A2063G	3	P1-1	T1-3R
SRR12190406	2019	China_Taiwan	Asia	A2063G	3	P1-1	T1-3R
SRR12190407	2019	China_Taiwan	Asia	non-MUT	17	P1-1	T1-2
SRR12190408	2018	China_Taiwan	Asia	A2063G	3	P1-1	T1-3R
SRR12190409	2018	China_Taiwan	Asia	A2063G	17	P1-1	T1-2
SRR12190410	2019	China_Taiwan	Asia	non-MUT	14	P1-2	T2-2
SRR12190411	2019	China_Taiwan	Asia	A2063G	3	P1-1	T1-3R
SRR12190412	2019	China_Taiwan	Asia	A2063G	3	P1-1	T1-3R

SRR12190413	2018	China_Taiwan	Asia	non-MUT	17	P1-1	T1-2
SRR12190414	2018	China_Taiwan	Asia	non-MUT	17	P1-1	T1-2
SRR12190415	2018	China_Taiwan	Asia	A2063G	17	P1-1	T1-2
SRR12190416	2018	China_Taiwan	Asia	A2063G	17	P1-1	T1-2
SRR3924583	2013	USA	North America	non-MUT	3	P1-1	T1-3
SRR3924584	2011	USA	North America	A2063G	20	P1-1	T1-3
SRR3924586	2012	USA	North America	A2063G	20	P1-1	T1-3
SRR3924594	2011	USA	North America	non-MUT	20	P1-1	T1-3
SRR3924595	2012	South_Africa	Africa	non-MUT	2	P1-2	T2-1
SRR3924601	1986	USA	North America	non-MUT	2	P1-2	T2-1
SRR3924603	2011	USA	North America	non-MUT	14	P1-2	T2-2
SRR3924607	1993	USA	North America	non-MUT	2	P1-2	T2-1
SRR3924604	1988	USA	North America	non-MUT	2	P1-2	T2-1
SRR3924611	1994	USA	North America	non-MUT	7	P1-2	T2-1
SRR3924612	2007	USA	North America	non-MUT	14	P1-2	T2-2
SRR3924613	1994	USA	North America	non-MUT	7	P1-2	T2-1
SRR3924614	1996	USA	North America	non-MUT	2	P1-2	T2-1
SRR3924615	2007	USA	North America	non-MUT	3	P1-1	T1-3
SRR3924616	1994	USA	North America	non-MUT	2	P1-2	T2-1
SRR3924618	2007	USA	North America	non-MUT	3	P1-1	T1-3
SRR3924619	1994	USA	North America	non-MUT	7	P1-2	T2-1
SRR3924620	1974	USA	North America	non-MUT	2	P1-2	T2-1
SRR3924621	2012	USA	North America	non-MUT	20	P1-1	T1-3
SRR3924622	2012	USA	North America	non-MUT	14	P1-2	T2-2
SRR3924623	2011	USA	North America	non-MUT	14	P1-2	T2-2
SRR3924624	2014	USA	North America	non-MUT	14	P1-2	T2-2
SRR3924625	2014	USA	North America	non-MUT	14	P1-2	T2-2
SRR3924626	2010	USA	North America	non-MUT	17	P1-1	T1-2
SRR3924627	2010	USA	North America	A2063G	20	P1-1	T1-3
SRR3924629	2013	USA	North America	non-MUT	14	P1-2	T2-2
SRR3924630	2013	USA	North America	non-MUT	3	P1-1	T1-3
SRR3924633	2013	USA	North America	non-MUT	3	P1-1	T1-3
SRR3924634	2013	USA	North America	non-MUT	2	P1-2	T2-1
SRR3924636	2012	USA	North America	A2063G	20	P1-1	T1-3
SRR3924637	2011	USA	North America	non-MUT	17	P1-1	T1-2
SRR3924638	2012	USA	North America	A2063G	20	P1-1	T1-3
SRR3924639	1992	Canada	North America	non-MUT	3	P1-1	T1-3
SRR3924640	2012	USA	North America	non-MUT	20	P1-1	T1-3

SRR3924641	2012	USA	North America	non-MUT	17	P1-1	T1-2
SRR3924642	2011	USA	North America	non-MUT	20	P1-1	T1-3
SRR3924643	2012	USA	North America	non-MUT	17	P1-1	T1-2
SRR3924644	2012	USA	North America	A2063G	20	P1-1	T1-3
SRR3924645	2011	USA	North America	non-MUT	20	P1-1	T1-3
SRR3924646	2012	USA	North America	non-MUT	20	P1-1	T1-3
SRR3924648	2010	Guatemala	North America	non-MUT	3	P1-1	T1-3
SRR3924649	2010	USA	North America	A2063G	20	P1-1	T1-3

Supplementary Table 3. Aisa-dominant genetic alterations and function.

Function category	Genotype	Position	Type	RefSite	SnpSite	*Gene*	Synonym	NTChange	AAChange	Function
Genome Stability	1	814696	SNV	C	T	*dnaA*	MPN_686	C92T	31:R>Q	chromosomal replication initiator protein DnaA
	2	37004	SNV	C	A	*polC*	MPN_034	C4128A	1376:M>I	DNA polymerase III (dnaE) alpha chain
	2	314199	SNV	C	A	*topA*	MPN_261	C1944A	648:N>K	DNA topoisomerase I
	2	426113	SNV	C	T	*lig*	MPN_357	C1399T	467:E>K	DNA ligase
	2	597298	SNV	G	T	*recA*	MPN_490	G529T	177:R>S	recombination protein RecA
	2	740343	SNV	C	T	*dnaX*	MPN_618	C2002T	668:E>K	DnaX
	2	413005^413006	Insertion	-	T	*hsdR*	MPN_345			type I restriction enzyme-like protein
Virulence Factor	1	30052	SNV	G	A	*rpoE*	MPN_024	G249A	83:W>*	DNA-directed RNA polymerase delta subunit
	1	262175	SNV	G	T	-	MPN_213	G47T	16:G>V	conserved hypothetical protein-P116
	1	635480	SNV	C	T	*rpoB*	MPN_516	C982T	328:E>K	RNA polymerase beta subunit
	2	30238	SNV	C	A	*rpoE*	MPN_024	C435A	145:N>K	DNA-directed RNA polymerase delta subunit
	2	506137	SNV	C	T	*alaS*	MPN_419	C287T	96:G>D	alanyl-tRNA synthetase
	2	546188	SNV	C	T	*hmw1*	MPN_447	C173T	58:G>E	cytadherence accessory protein HMW1
INFORMATION STORAGE AND PROCESSI	1	288661	SNV	G	A	-	MPN_236	G1274A	425:S>N	Glu-tRNA amidotransferase subunit C
	2	218822	SNV	G	A	*rplC*	MPN_165	G853A	285:E>K	ribosomal protein L3
	2	223494	SNV	C	A	*rpsQ*	MPN_174	C124A	42:H>N	ribosomal protein S17
	2	327817	SNV	C	T	*lysS*	MPN_277	C962T	321:T>I	lysyl-tRNA synthetase

NG	2	484390	SNV	G	A	*proS*	MPN_402	G862A	288:D>N	putative prolyl-tRNA synthetase
	2	675993	SNV	C	G	*argS*	MPN_556	C1312G	438:A>P	arginyl-tRNA synthetase
	2	671707	SNV	C	A	-	MPN_551	C542A	181:S>I	conserved hypothetical protein
	1	39428	SNV	G	A	*polC*	MPN_034	G1704A	568:D>D	DNA polymerase III (dnaE) alpha chain
	1	457780	SNV	T	C	*fpg*	MPN_380	T634C	212:L>L	formamidopyrimidine-DNA glycosylase
	1	585596	SNV	C	T	*valS*	MPN_480	C324T	108:A>A	valyl-tRNA synthetase
	1	802632	SNV	G	A	*gltX*	MPN_678	G816A	272:Y>Y	glutamyl-tRNA synthetase
	2	24077	SNV	G	A	*yb95*	MPN_020	G1056A	352:K>K	helicase-like protein
	2	136884	SNV	C	T	*pheT*	MPN_106	C126T	42:N>N	phenylalanyl-tRNA synthetase beta chain
	2	156913	SNV	G	A	*parB*	MPN_122	G447A	149:K>K	topoisomerase IV subunit B
	2	407594	SNV	T	C	*mutB1*	MPN_341	T2022C	674:R>R	DNA helicase II
	2	414692	SNV	G	A	*hsdR*	MPN_347	G708A	236:E>E	type I restriction enzyme-like protein
	2	613114	SNV	C	A	-	MPN_504	C375A	125:S>S	hypothetical protein
	2	786699	SNV	G	T	-	MPN_663	G564T	188:L>L	conserved hypothetical protein
	2	204764	SNV	C	T	-	MPN_153	C2382T	794:N>N	conserved hypothetical protein
	2	205403	SNV	G	A	-	MPN_153	G3021A	1007:E>E	conserved hypothetical protein
CELLULAR PROCESSES AND SIGNALING	1	806664	SNV	G	A	*devA*	MPN_683	G227A	76:P>L	ABC transporter subunit
	1	60570	SNV	G	A	-	MPN_049	G952A	318:V>I	membrane export protein family
	1	328571	SNV	G	T	*yefE*	MPN_278	G287T	96:R>L	udp-galactopyranose mutase
	1	738547	SNV	T	G	*hsdS*	MPN_615	T448G	150:K>Q	hypothetical protein
	2	21006	SNV	C	A	*pmd1*	MPN_018	C1682A	561:P>Q	transport ATP-binding protein
	2	26209	SNV	C	T	*dnaJ*	MPN_021	C50T	17:S>F	heat shock protein DnaJ
	2	47290	SNV	C	T	-	MPN_039	C97T	33:Q>*	conserved hypothetical protein
	2	50507	SNV	C	T	-	MPN_042	C1216T	406:R>C	conserved hypothetical protein
	2	350256	SNV	C	T	*lsp*	MPN_293	C103T	35:G>S	prolipoprotein signal peptidase
	2	513897	SNV	G	A	-	MPN_426	G646A	216:R>C	SMC family, chromosome/DNA binding/protecting functions
	2	807575	SNV	C	T	-	MPN_684	C4966T	1656:V>I	conserved hypothetical protein
	1	809277	SNV	C	T	-	MPN_684	C3264T	1088:L>L	conserved hypothetical protein
	2	22997	SNV	C	T	*msbA*	MPN_019	C1890T	630:R>R	transport ATP-binding protein

	2	59939	SNV	C	A	-	MPN_049	C321A	107:L>L	membrane export protein family
	2	97927	SNV	C	T	-	MPN_080	C1521T	507:N>N	Probably ABC transporter membrane protein subunit
	2	257719	SNV	C	T	*secA*	MPN_210	C480T	160:N>N	preprotein translocase SecA
	2	276188	SNV	C	T	*lgt*	MPN_224	C690T	230:N>N	prolipoprotein diacylglyceryl transferase
	2	474577	SNV	T	C	-	MPN_396	T1890C	630:L>L	SecD-like protein
	2	510665	SNV	G	A	*ftsY*	MPN_425	G924A	308:N>N	cell division protein FtsY
	2	511127	SNV	G	T	*ftsY*	MPN_425	G462T	154:A>A	cell division protein FtsY
	2	512518	SNV	A	G	-	MPN_426	A2025G	675:N>N	SMC family, chromosome/DNA binding/protecting functions
	2	678762	SNV	G	A	*gidA*	MPN_557	G1431A	477:L>L	NADH-binding oxidoreductase GidA
	2	785104	SNV	G	A	-	MPN_661	G1035A	345:D>D	conserved hypothetical protein
	2	794965	SNV	G	A	*ftsH*	MPN_671	G1467A	489:Y>Y	cell division protein FtsH
	2	184739	SNV	A	G	-	MPN_141	A3882G	1294:V>V	adhesin P1
	2	188458	SNV	C	T	-	MPN_142	C2712T	904:V>V	cytadherence protein
METABOLISM	1	468455	SNV	T	C	*pdhC*	MPN_391	T601C	201:T>A	dihydrolipoamide acetyltransferase component (E2)
	1	602203	SNV	C	T	*yjfS*	MPN_496	C1525T	509:A>T	phosphotransferase protein-like protein
	1<br>1	560773.560775<br>560772^560773	Delation<br>Insertion	CAC<br>-	-<br>(CAC)n	-<br>-	MPN_459			conserved hypothetical protein
	2	79823	SNV	A	G	*deoC*	MPN_063	A71G	24:D>G	deoxyribose-phosphate aldolase
	2	123900	SNV	G	A	-	MPN_095	G611A	204:R>H	amino acid permease
	2	211534	SNV	A	G	*yaaC*	MPN_158	A388G	130:I>V	riboflavin kinase/FMN adenylyltransferase
	2	241098	SNV	C	T	-	MPN_199	C1682T	561:S>F	conserved hypothetical protein
	2	275257	SNV	G	A	-	MPN_223	G687A	229:M>I	HPr(Ser) kinase
	2	466215	SNV	C	T	*lplA*	MPN_389	C232T	78:A>T	lipoate protein ligase
	2	720387	SNV	A	G	*atpD*	MPN_598	A589G	197:Y>H	ATP synthase beta chain
	1	798553	SNV	C	T	*ldh*	MPN_674	C735T	245:L>L	L-lactate dehydrogenase
	2	82281	SNV	T	C	*cpsG*	MPN_066	T201C	67:A>A	CpsG
	2	229456	SNV	A	G	*adk*	MPN_185	A507G	169:V>V	adenylate kinase

	2	240844	SNV	T	C	-	MPN_199	T1428C	476:D>D	conserved hypothetical protein
	2	379596	SNV	T	G	*gap1*	MPN_319	T939G	313:G>G	general amino acid permease GAP1-like protein
	2	467904	SNV	G	A	*pdhC*	MPN_391	G1152A	384:R>R	dihydrolipoamide acetyltransferase component (E2)
	2	472589	SNV	T	C	*nox*	MPN_394	T327C	109:V>V	NADH oxidase
	2	699516	SNV	G	A	*glyA*	MPN_576	G390A	130:Y>Y	serine hydroxymethyltransferase
	2	728194	SNV	G	A	*phoU*	MPN_608	G553A	185:L>L	phosphate transport system regulatory protein
	2	200338	SNV	T	C	-	MPN_152	T516C	172:N>N	conserved hypothetical protein
	2	417436	SNV	G	A	*ygiH*	MPN_350	G675A	225:H>H	conserved hypothetical protein
RNA Coding	1	122119	SNV	A	G	-	MPN_r02	A2063G	688:E>G	23S ribosomal RNA
	2	571926	SNV	G	A	-	MPN_s04	G102A	34:K>K	10sa RNA
POORLY CHARACT ERIZED	1	351200	SNV	G	T	-	MPN_295	G135T	45:K>N	conserved hypothetical protein
	1	446618	SNV	C	T	-	MPN_373	C124T	42:E>K	hypothetical protein
	1	479364	SNV	G	T	-	MPN_399	G824T	275:P>Q	conserved hypothetical protein
	1	489535	SNV	C	T	-	MPN_407	C613T	205:E>K	lipase
	1	549877	SNV	C	T	*come3*	MPN_451	C746T	249:S>N	competence locus operon protein 3-like protein
	1	734674	SNV	G	A	-	MPN_612	G1397A	466:S>F	conserved hypothetical protein
	1	781478	SNV	C	T	-	MPN_656	C433T	145:V>I	GTP-binding protein
	1	789899	SNV	C	T	-	MPN_666	C490T	164:P>S	conserved hypothetical protein
	1	197250	SNV	C	A	-	MPN_149	C401A	134:T>N	hypothetical protein
	2	142962	SNV	A	T	-	MPN_110	A602T	201:D>V	conserved hypothetical protein
	2	143549	SNV	C	T	-	MPN_110	C1189T	397:Q>*	conserved hypothetical protein
	2	306434	SNV	G	A	-	MPN_256	G122A	41:S>N	conserved hypothetical protein
	2	306674	SNV	C	T	-	MPN_256	C362T	121:T>I	conserved hypothetical protein
	2	351095	SNV	A	G	-	MPN_295	A30G	10:*>W	conserved hypothetical protein
	2	487033	SNV	G	A	-	MPN_405	G513A	171:W>*	conserved hypothetical protein
	2	532056	SNV	C	T	-	MPN_439	C607T	203:D>N	Mollicute specific lipoprotein
	2	540217	SNV	C	A	-	MPN_444	C1523A	508:S>I	conserved hypothetical protein
	2	690768	SNV	C	T	*spg*	MPN_568	C661T	221:A>T	small GTPase ERA involved in regulating

										metabolism and cell division
	2	762164	SNV	G	A	-	MPN_635	G661A	221:A>T	conserved hypothetical protein
	2	171593	SNV	G	A	-	MPN_132	G545A	182:G>D	hypothetical protein
	2	185212	SNV	A	G	-	MPN_141	A4355G	1452:N>S	adhesin P1
	2	440120	SNV	G	T	-	MPN_369	G536T	179:T>K	conserved hypothetical protein
	2	495880	SNV	G	T	-	MPN_411	G161T	54:T>K	conserved hypothetical protein
	2	315058	SNV	G	A	-	MPN_262	G678A	226:L>L	conserved hypothetical protein
	2	579522	SNV	G	A	-	MPN_475	G1236A	412:S>S	GTPase
	2	171558	SNV	G	T	-	MPN_132	G510T	170:G>G	hypothetical protein
	2	716346	SNV	G	T	-	MPN_593	G120T	40:T>T	conserved hypothetical protein
	1	538723^ 538724	Insertion	-	(TAC)n	-	MPN_444	-	1006:(M)n	conserved hypothetical protein
	2	113356	Deletion	A	-	-	MPN_090	-	-	adhesin P1 (group2) homolog
	2	735397	Deletion	A	-	-	MPN_612	-	-	conserved hypothetical protein
	2	35991	Insertion	-	T	-	MPN_032	-	-	hydrolase
	2	565850^ 565851	Insertion	-	TGG	-	MPN_462	-	254:T	hypothetical protein
	2	565852.5 65853	Deletion	CA	-	-		-	-	
	2	565858.5 65859	Deletion	CA	-	-		-	-	
	2	565861^ 565862	Insertion	-	A	-		-	-	
	2	765798.7 65807	Deletion	GCATC TTTTT	-	-	MPN_639	-	-	conserved hypothetical protein

1.2 Supplementary Figures

Supplementary Figure 1. Phylogenetic tree based on genome-wide SNPs of the 160 newly sequenced strains along with 430 *M. pneumoniae* genomes available from public domain.

A maximum-likelihood phylogenetic tree constructed by RAxML with the GTRgamma substitution model. The tree was rooted on midpoint. Outer strips from inner to outer are color coded by ST groups, 23S rRNA mutation, isolation year and continent (key). The tree scale represents substitutions per site.

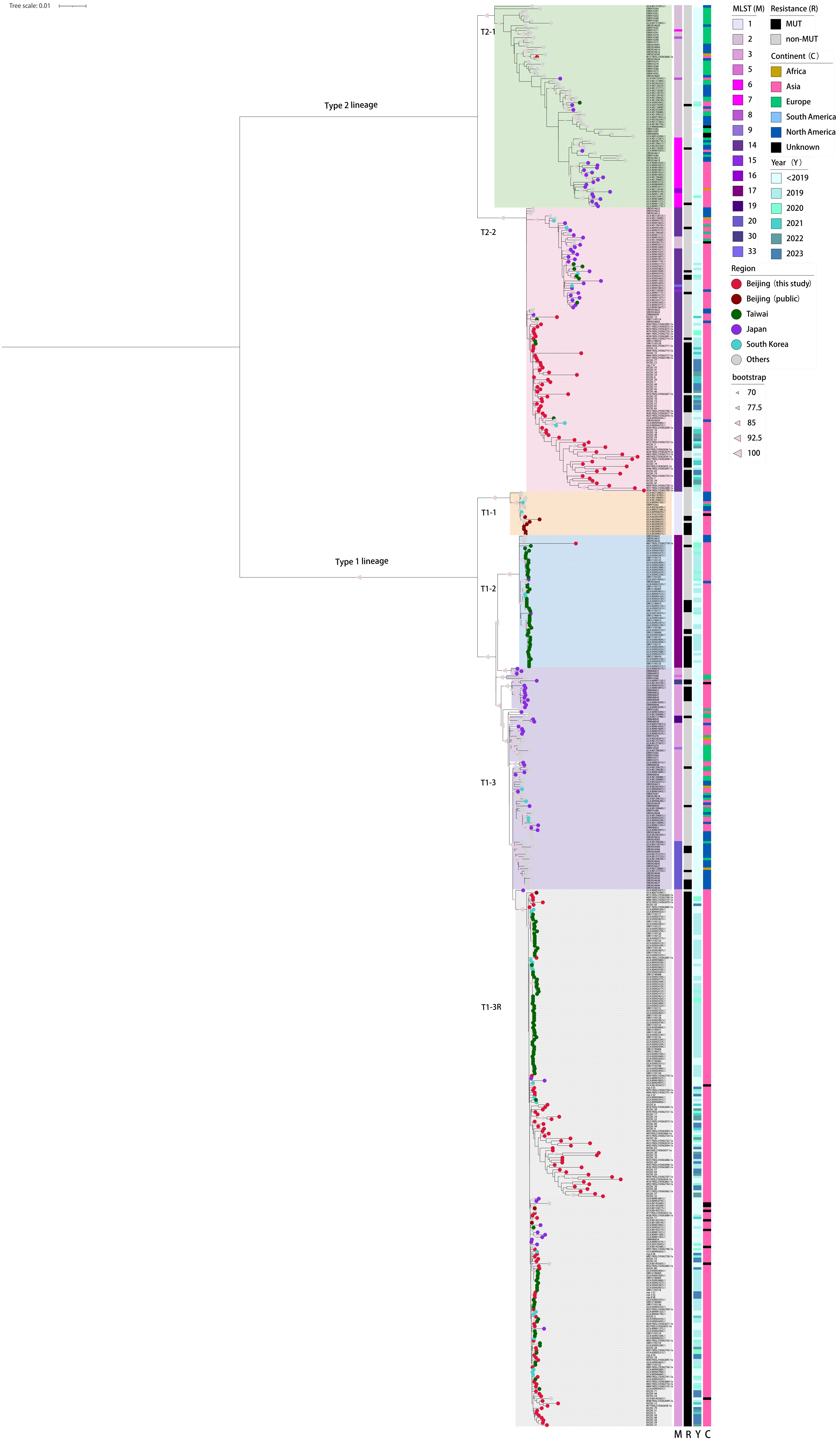

Tree scale: 0.01
Type 2 lineage
Type 1 lineage
T2-1
T2-2
T1-1
T1-2
T1-3
T1-3R
M R Y C
MLST (M)
1
2
3
5
6
7
8
9
14
15
16
17
19
20
30
33
Resistance (R)
MUT
non-MUT
Continent（C）
Africa
Asia
Europe
South America
North America
Unknown
Year （Y）
<2019
2019
2020
2021
2022
2023
Region
Beijing（this study）
Beijing（public）
Taiwai
Japan
South Korea
Others
bootstrap
70
77.5
85
92.5
100
